# From sensory to perceptual manifolds: The twist of neural geometry

**DOI:** 10.1126/sciadv.adv0431

**Published:** 2025-12-10

**Authors:** Heng Ma, Longsheng Jiang, Tao Liu, Jia Liu

**Affiliations:** ^1^ Department of Psychological and Cognitive Sciences & Tsinghua Laboratory of Brain and Intelligence, Tsinghua University, Beijing 100084, China.; ^2^ Beijing Academy of Artificial Intelligence (BAAI), Beijing 100085, China.

## Abstract

Classification constitutes a fundamental cognitive challenge for both biological and artificial intelligence systems. Here, we investigated how the brain categorizes stimuli that are not linearly separable in the physical world by analyzing the geometry of neural manifolds formed by macaque V2 neurons during a classification task involving motion-induced illusory contours. We identified two related but distinct neural manifolds: the sensory and perceptual manifolds. The sensory manifold was embedded in a three-dimensional subspace defined by three stimulus features, where contour orientations remained linearly inseparable. However, through a sequence of geometric transformations equivalent to twist operations, this three-dimensional sensory manifold expanded into a seven-dimensional perceptual manifold, enabling the linear separability of contour orientations. Computational modeling further demonstrated that this dimension expansion was facilitated by neurons exhibiting nonlinear mixed selectivity with heterogeneous response profiles. These findings provide insights into how biological neural networks enhance the dimensionality of representational spaces, illuminating the geometric mechanism underlying the transformation from sensation to perception.

## Teaser

The brain uses geometric twists to expand neural dimensionality, thus untangling perception from sensation.

## INTRODUCTION

Imagine a person trying to identify various objects such as a cup, a book, and a pen on a clustered desk. The brain must process complex and overlapping sensory inputs with variations in light, angles, and occlusions by neurons encoding different features of the objects and their combinations to classify ultimately each object accurately (*1*–*3*). One primary challenge in this classification task lies in the prevalence of linearly inseparable problems, where perfectly segregating data points into their respective classes using a linear boundary is infeasible (*4*, *5*). Machine learning algorithms often resort to complex, nonlinear decision boundaries to address this issue, using techniques such as kernel methods and deep learning (*6*, *7*). Here, we asked how the brain addresses linearly inseparable problems present in the physical world from the perspective of neural geometry (*8*–*10*) constituted by the collective activity of large groups of neurons, an approach recently applied to various domains such as vision (*11*, *12*), memory (*13*, *14*), decision (*15*–*17*), navigation (*18*, *19*), and motor execution (*20*–*23*) to explore their characteristics in a high-dimensional neural space.

**Fig. 1.**
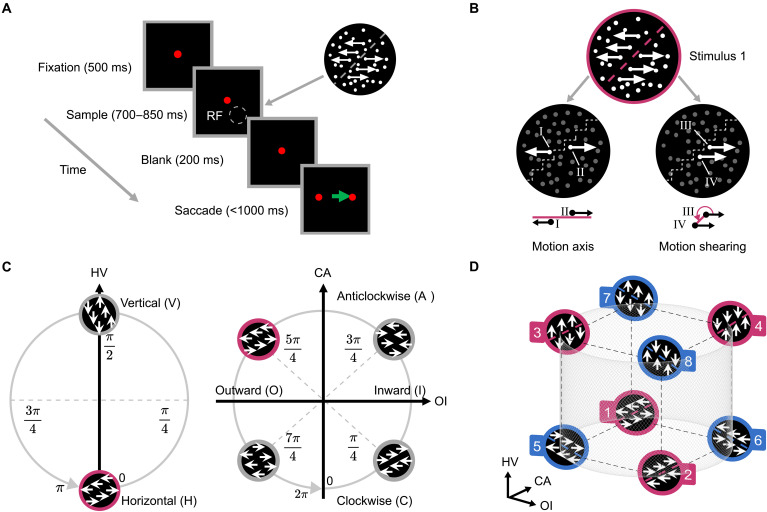
Stimulus space. (**A**) Left: Schematic of the discrimination task. Right: Example of a right-tilted contour orientation formed by surrounding moving dots. Modified from [(*32*) changes include: Refined the borders of each event-window schematic, explicitly indicated the time range of the sample event window, adjusted the position of the receptive-field schematic, added additional arrows to the moving dots in the stimulus example, and added an arrow from the stimulus example to the spatial location at which the stimulus was presented (http://creativecommons.org/licenses/by/4.0/)]. RF: array population receptive field. (**B**) Example stimulus characterized by the motion axis and sheared configuration. (**C**) Stimulus feature axes. Left: HV axis. Right: OI and CA axes. The red circle highlights a horizontal, outward, and anticlockwise-like stimulus. (**D**) 3D stimulus space. The eight stimuli used in the experiment, numbered and color-coded by contour orientation (red: right-tilted; blue: left-tilted).

To do this, we first designed a set of stimuli that cannot be linearly classified along one stimulus feature or linear combination of stimulus features. Specifically, we used a visual illusion of motion-induced contours [MICs; (*24*–*26*)] (Fig. 1B). MIC is a second-order contour, similar to other well-documented illusory contours, such as contrast-defined contours (*27*), disparity-defined contours (*28*, *29*), and texture-defined contours (*30*). This stimulus is not defined by luminance edges but arises mostly from the coherent spatial-temporal patterns of dot motion. There are three independent stimulus features of moving dots that are more basic. They determine a particular instance of MIC stimuli (*26*, *31*, *32*): Dots move (i) either horizontally or vertically, (ii) outwardly or inwardly, and (iii) clockwise-like or anticlockwise-like (Fig. 1C). Accordingly, a three-dimensional (3D) stimulus space is thus constructed (Fig. 1D), with each axis corresponding to a stimulus feature of moving dots. Although each stimulus feature of moving dots is linearly separable, the orientations of illusory boundaries, constructed by combining these three features, become linearly inseparable in this stimulus space. That is, MIC stimuli sharing the same contour orientation (e.g., right-tilted, red) are interspersed among those with the opposing orientation (i.e., left-tilted, blue; see detailed explanation in Results). This shows that the classification of contour orientations presents a linearly inseparable problem in the physical world.

In the mental world, however, the linear classification of contour orientations becomes feasible, as previous neurophysiological studies have revealed that single neurons in macaques’ V2 exhibit selectivity for cue-invariant contour orientations (*24*, *32*). Accordingly, we analyzed neuronal activity data recorded in the V2 area (*32*), aiming to elucidate how the linearly inseparable problem in the stimulus space becomes linearly separable in the high-dimensional neural space constructed by the collective activity of V2 neurons. According to the contours that do not exist in the physical world and being created by the brain when integrating individual motions and interpreting symmetrical movement patterns, we examined two types of neural manifolds embedded in this high-dimensional space: the sensory and perceptual manifolds. The sensory manifold, which arises from the sensation process, directly responds to physical stimuli without involving interpretation and provides the raw sensory data that the brain uses to build perceptual experiences. Given its correspondence to external stimuli, contour orientations likely remain linearly inseparable in the sensory manifold. In contrast, the perceptual manifold reflects the brain’s effort to interpret and make sense of the sensory data, where neural states for MIC stimuli may be separately clustered on the basis of contour orientations. Therefore, understanding the geometric difference between the sensory and perceptual manifolds and further deriving potential mechanisms that create such a difference provide a mechanistic perspective on how perception arises from sensation.

## RESULTS

### Stimulus space and linearly inseparable problems

Two macaque monkeys were trained to perform a classification task by executing a saccade toward the side corresponding to the orientation of a second-order MIC. The MIC stimuli used in this study were generated by moving dots in a circular viewing area where dots in opposing half areas moved in opposite directions (Fig. 1A).

Using two parameters describing motion patterns and spatial arrangement, we could systematically determine the MIC stimuli. One parameter was the motion axes. As illustrated in Fig. 1B, dots in opposing half areas moved in opposite directions along a motion axis (e.g., dot I and dot II in Fig. 1B, left). The motion axis could rotate within a range of [ 0,π ], creating a circular structure (fig. S1A). The other parameter was the sheared configuration. It quantified the angular location of one dot (e.g., dot IV in Fig. 1B, right) relative to the adjacent other (e.g., dot III), with respect to their moving direction. This configuration was especially salient at locations where the two halves of the coherently moving dots met (fig. S2, A and B). The sheared configuration varies periodically over [ 0,2π ] (fig. S1B). Given the values of the motion axis and sheared configuration, we determined an MIC orientation in the range [ 0,π].

Accordingly, these two periodic parameters together define a stimulus manifold with a torus topology (fig. S3, left), where each point represents a stimulus generated by a unique combination of the motion axis and sheared configuration. In this study, we only used a subset of this stimulus manifold. Specifically, we first projected the 2D circular structure of the motion axis onto the 1D axis, which included values of 0 (horizontal) and π/2 (vertical; Fig. 1C, left). This 1D axis was referred to as the HV (horizontal versus vertical) dimension. We then retained the 2D circular structure of the sheared configuration (Fig. 1C, right), with the two dimensions, respectively, referred to as OI (outward versus inward) and CA (clockwise-like versus anticlockwise-like), according to the appearance of the stimuli (fig. S2C). Consequently, the subset stimulus manifold has a cylindrical topology, embedded in the stimulus space spanned by the HV, OI, and CA dimensions (Fig. 1D and fig. S3, right).

Eight MIC stimuli were sampled from the stimulus space to cover two conditions in each dimension (Fig. 1D; see movie S1 for the eight stimuli). These MIC stimuli had two orientations, either right-tilted ( π/4 , no. 1 to no. 4, red) or left-tilted ( 3π/4 , no. 5 to no. 8, blue). The stimuli of opposite orientations interspersed with each other, resulting in MIC orientations being linearly inseparable within this stimulus space. However, both macaques adeptly performed the task with an accuracy exceeding 90% at 100% motion coherence (*32*). Next, we investigated how neurons in macaques’ V2 tackle this linearly inseparable problem particularly from the perspective of neural geometry.

### Sensory and perceptual manifolds

**Fig. 2.**
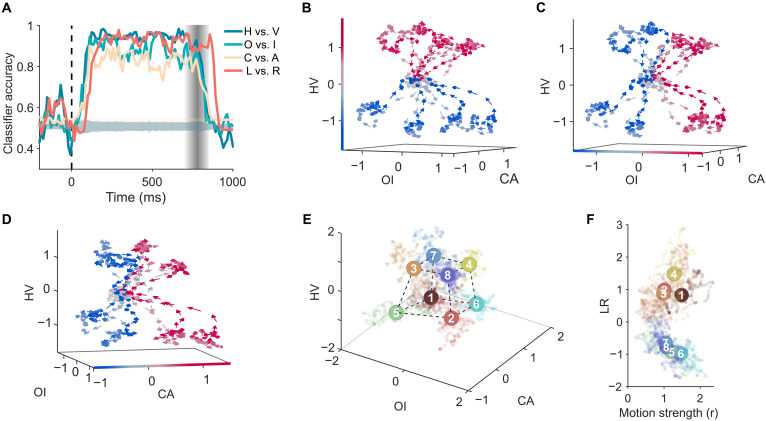
Neural representation of stimulus features and contour orientations. (**A**) Time-resolved classification accuracy for different stimulus features (HV, OI, and CA) and contour orientation (LR). Shaded gray area denotes chance-level performance estimated by shuffled control (mean ± 3 SDs). Vertical dashed lines mark stimulus onset (0 ms). Vertical graded regions mark stimulus offset (randomly in 700 to 850 ms for different trials). (**B** to **D**) Dynamic trajectories of neural states projected onto the neural subspace defined by the HV (B), OI (C), and CA (D) axes. Lighter colors indicate earlier time points; more saturated colors indicate later time points. Arrows indicate the direction of trajectory evolution. Neural states prior to stimulus onset are marked in gray. Blue and red colors indicate categorizations along the HV, OI, and CA axes, respectively. The motion coherence level shown here is 7. (**E**) Sensory manifold. The point cloud centers of the eight stimuli in the steady phase (200 to 500 ms). Dashed lines highlight geometric structure of the centers. Cool and warm colors indicate the two contour orientations. All motion coherence levels were used. (**F**) Projection of population neural activity onto the task-relevant decision axis (L versus R orientation discrimination). Same color code as in (E). Motion strength ( r ) quantifying Euclidean distance between the neural states and the origin in (E) is included for display purposes only.

To investigate how the brain addressed this linearly inseparable problem, we used neural activity data from V2 neurons of two monkeys (93 neurons in total) to construct a high-dimension neural space (see Materials and Methods; for neurons’ receptive fields and selectivity, see fig. S4). First, we used linear support vector machines (SVMs) to analyze the collective activity to all the eight stimuli to determine whether the three feature axes of the stimulus space (Fig. 1D)—HV ( {1,2,5,6} versus {3,4,7,8} ), OI ( {1,3,5,7} versus {2,4,6,8} ), and CA ( {2,3,5,8} versus {1,4,6,7})—could be decoded. We found that classifications along all three axes were linearly separable, with accuracies above 75% and significantly higher than the shuffled baseline (bootstrap *t* tests, all Ps < 0.001) (Fig. 2A). This finding was replicated with other methods such as the analysis on population average responses and the principal components analysis (PCA) (fig. S5). Critically, the optimal separation direction vectors (determined by SVM, see Materials and Methods) for HV, OI, and CA were mutually orthogonal. That is, the distribution of the subtended angles between these vectors was significantly more concentrated around 90° than the distribution of angles between two random direction vectors and also significantly larger than angle distributions between the same classification vectors (fig. S6).

To visualize the dynamic encoding of the stimuli by the neuronal population, we projected the neural states in the high-dimensional neural space into a 3D neural subspace formed by the direction vectors for HV, OI, and CA (see Materials and Methods). Figure 2B shows that, before stimulus onset, neural states for the stimuli were closely clustered and inseparable. Following the onset, these states gradually spread and became completely separable along the HV axis. They then remained in a steady phase for the duration of stimulus presentation before returning to their original locations after stimulus offset (see movie S2 and fig. S11). Similar dynamic patterns were observed for the OI (Fig. 2C) and CA (Fig. 2D) axes as well. To further illustrate the neural geometry constructed by the neural states at the steady phase, we used the activity magnitude of neurons from 200 to 500 ms poststimulus onset as the neural states, which were then projected to this 3D subspace (see Materials and Methods). Figure 2E shows that the neural states were located at eight vertices of a slightly distorted cube, corresponding to the geometric relation among stimuli in the original stimulus space (Fig. 1D). This neural manifold embedded in the high-dimensional neural space is referred to as the “sensory manifold” because it directly responded to the stimulus space without involving interpretation, as contour orientations remain linearly inseparable in this manifold. To further demonstrate that this manifold was associated with sensory processing, we examined the relationship between its geometry and the intensity of sensory signals. We found that the sensory manifold was sensitive to variations in the coherence of moving dots in the stimuli, giving rise to a series of concentric sensory manifolds, with higher coherence levels eliciting manifolds of larger size (fig. S8). Together, the sensory manifold faithfully represented the stimulus space, with HV, OI, and CA serving as the axes of the neural subspace that embedded this manifold.

Based on previous studies showing that individual neurons that are sensitive to contour orientation (*24*, *32*), we used the SVM analysis to explore axes in this high-dimensional neural space that could linearly decode contour orientations. We identified an axis that can differentiate left-tilted orientation from the right-tilted orientation, referred to as the LR axis, with an accuracy comparable to that of decoding the stimuli’s features (i.e., HV, OI, and CA) (Fig. 2A, orange line). Figure 2F illustrates the neural states along the LR axis, where contour orientations became linearly separable (for dynamic trajectories, see fig. S11 and movie S2), consistent with the finding from studies on single neurons (*24*, *32*). Furthermore, this LR axis was orthogonal to the three stimulus feature axes of the sensory manifold (fig. S6A), suggesting that the LR axis emerged not directly from the stimuli but from the interpretation of the sensory data. Evidence supporting this conjecture comes from the analysis on the latency of the emergence of the LR axis, which occurred about 30 ms after the emergence of the axes encoding the stimuli’s features (i.e., HV, OI, and CA) (Fig. 2A). This observation aligns with previous findings on visual motion segregation (*24*, *26*, *33*–*35*), implying that extra time may be needed to form this new axis. This new neural manifold, embedded in the high-dimensional neural space including the LR axis for representing contour orientations and the axes for stimulus features (i.e., HV, OI, and CA), is referred as the perceptual manifold, where the linearly inseparable problem was addressed. Next, we explored how this LR axis was formed through geometric transformation.

### NMS neurons and twist operations

Traditional solutions to linearly inseparable problems such as the exclusive-or (XOR) problem involve expanding dimensions of the original representational space (*4*, *5*). Previous studies have shown that neurons with nonlinear mixed selectivity (NMS) can expand dimensions by responding to combinations of input features (*5*, *36*–*38*) and using nonlinear activation functions (*39*, *40*) to capture higher-order interactions. All the V2 neurons analyzed in this study showed interactive responsiveness to the three stimulus features (significant three-way interactions: minimum $F_{1,2200}=18.25$ , P<0.001 ), consistent with the characteristic response patterns of nonlinear mixed selective neurons previously identified by analysis of variance (ANOVA)–based methods (*38*, *41*). These findings suggest that these NMS neurons in the V2 area may contribute to generating the LR dimension.

**Fig. 3.**
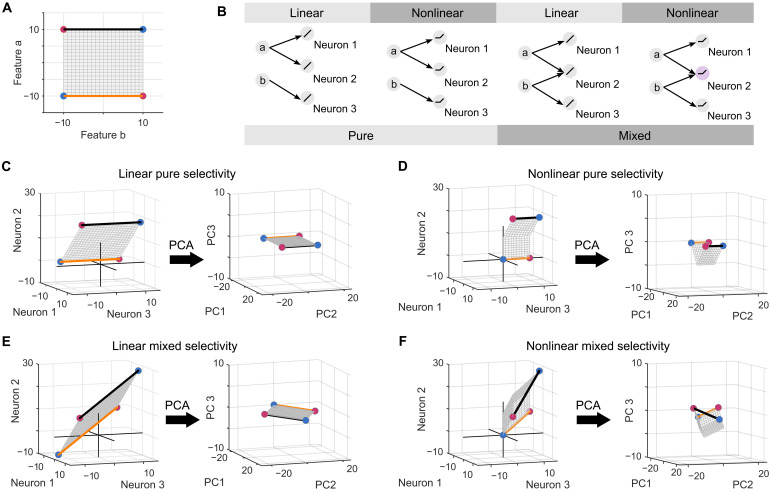
NMS neurons and twist operations. (**A**) 2D feature sheet represents possible combinations of stimulus features a and b . Four corners are colored red and blue to indicate different categories. Two connecting edges are highlighted in orange and black. (**B**) Four types of neurons’ response profiles derived from the combination of input (pure versus mixed) and activation functions (linear versus nonlinear). The neuron marked in light purple denotes an NMS neuron. (**C** to **F**) Left: The geometries in the neural space constructed by (C) three linear pure selectivity neurons, (D) three nonlinear pure selectivity neurons, (E) two linear pure selectivity neurons and one linear mixed selectivity neuron, or (F) two nonlinear pure selectivity neurons and one NMS neuron. Right: The same geometries transformed into the PCs obtained from PCA of the four corner points.

To reveal how NMS neurons expand the dimensionality of representational spaces, we examined this phenomenon from two complementary yet interconnected perspectives: (i) a neural network–based (implementation-level) perspective and (ii) a mathematical (algorithmic-level) perspective. From the neural network perspective, we explicitly decomposed NMS of neurons into two distinct operations—linear mixing and nonlinear transformation—performed by the network connections. We examined how each step contributes to dimensional expansion of the representations. We used a minimalist one-layer network consisting of three neurons, $s_{1}$ , $s_{2}$ , and $s_{3}$ . These neurons receive inputs consisting of two continuous features, a and b , that form a 2D feature sheet (Fig. 3A). In this context, the XOR problem is defined as categorizing points located at diagonally opposite corners (i.e., red versus blue points). The neurons in this network (i) receive either pure (either a or b ) or mixed (the combination of a and b ) inputs, and (ii) have either linear or nonlinear (e.g., rectified linear unit, i.e., ReLU) activation functions, leading to four distinct output patterns (Fig. 3B).

Neurons with pure selectivity and the linear activation function (i.e., linear pure selectivity) afford only a simple affine transformation, including rotation and shifting, of the original feature sheet (Fig. 3C, left). Because the rotation axis is aligned with the orange and black edges, the edges remain parallel after the transformation. The parallel edges are more obvious in the principal component (PC) space obtained by PCA of the four corner points (Fig. 3C, right). For neurons with nonlinear pure selectivity, the feature sheet undergoes bending due to the nonlinear activation function (Fig. 3D, left). This bending occurs along an axis parallel to the orange and black edges, so the edges remain parallel after bending, and the four corner points still lie on the same plane as evidenced in the PC space (Fig. 3D, right). For neurons with linear mixed selectivity (Fig. 3E, left), the feature sheet experiences a complex affine transformation, with the rotation axis not aligning with the three main axes. However, even after this rotation, the edges remain parallel (Fig. 3E, right). In these three scenarios, neither bending nor rotation alone suffices to make the red and blue points linearly separable.

In contrast, NMS neurons perform both bending and rotation operations on the feature sheet, making the orange and black edges no longer parallel (Fig. 3F, left). When displaying it in the PC space (Fig. 3F, right), it becomes more obvious that the orange and black edges appear to be twisted from their original parallel configuration. The twisting creates a new axis perpendicular to the original orange and black edges. In this newly constructed representational space, the XOR problem becomes linearly separable. Here, the combined effects of bending and rotation operations equalize a “twist” operation. Accordingly, we refer to this characteristic of NMS neurons acting on the geometry of input features as the twist operation [for a similar idea, see (*42*)].

**Fig. 4.**
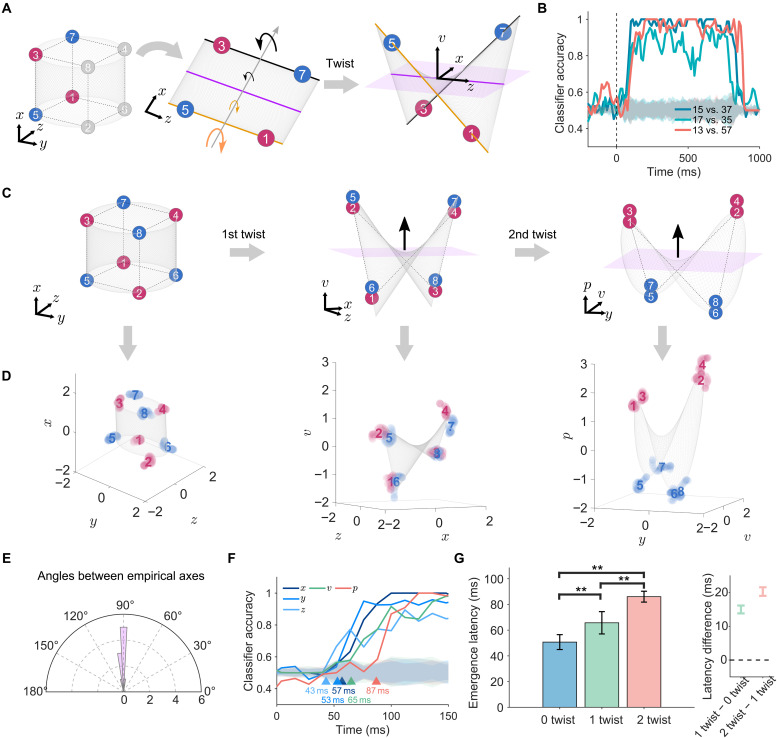
Twist operations on the classification of contour orientations. (**A**) Illustration of a twist operation on planar XOR problems. Left: Coplanar vertices {1,3,5,7} are colored on the basis of contour orientations, forming a standard XOR problem on the z-x plane. Middle: A twist operation around the x axis resolves the XOR problem by rotating two parallel edges in opposite directions. Right: This twist operation generates the v axis orthogonal to the z-x plane. (**B**) SVM classification accuracy over time. Shaded gray region (mean ± three SDs) denotes chance-level performance (shuffled control). (**C**) Illustration of double twist operations on a cubic XOR problem. Left: Theoretical sensory manifold in the x-y-z space. Middle: Intermediate manifold achieved after one twist operation on the z-x plane, with the emergence of the v axis. Right: Task-relevant p axis emerges after the second twist operation on the y-v plane. (**D**) Neural manifolds corresponding to the theoretical manifolds achieved through twist operations. The centers of the eight clouds of neural states were used to depict the geometry of neural manifolds through linear fitting. (**E**) Angle distribution among x, y, z, v, and p axes. (**F**) Emergence latencies of the z, y, x, v, and p axes. Triangles indicate averaged classification accuracies consistently exceed their corresponding baselines. Shaded gray regions denote the chance-level performance (shuffle control). (**G**) Left: Mean emergence latencies of the sensory axes (0-twist, blue, *N* = 300), the intermediate axes (1-twist, green, *N* = 300), and the contour axis (2-twist, red, *N* = 100). **: bootstrapped *t* test P<0.001 . Right: The 95% bootstrapped confidence intervals of the latency differences between axes of two different categories.

Building on this implementation-level explanation, we further sought a mathematical description of neurons’ NMS that concisely expresses both the mixing operation and nonlinear transformation on the stimulus features. Inspired by the multiplicative terms used to represent factor interaction in ANOVA (*43*), we observed the correspondence between the quadratic terms in algebra and the twist operation in geometry. Specifically, we used x , y , and z to denote the three original feature dimensions (HV, OI, and CA, respectively). The four vertices {1,3,5,7} in the z–x plane exemplify a standard planar XOR problem (Fig. 4A, left). The quadratic product v=zx describes the geometric twist operation on the z–x plane (Fig. 4A, middle). That is, it transforms the original 2D z–x plane into a curved surface in a 3D space (Fig. 4A, right), creating a new, orthogonal axis v . Thus, this quadratic expression succinctly captures the core geometric transformation, enabling solutions to XOR-type problems.

The quadratic product also corresponds to the XOR operation in logic. When letting the two levels on one axis represent the true and false values of a Boolean variable, the axes are equivalent to Boolean variables (see text S11). Also, the Boolean variable V of the v axis is equal to the XOR operation of the Boolean variables Z of the original z axis and X of the original x axis, expressed as V=Z⊕X . Therefore, the twist operation in neural geometry equals the XOR operator in logic, further explaining its efficacy in solving XOR problems.

To examine whether this theoretically derived v axis was actually present in the high-dimensional neural space, we trained linear SVMs to classify three sets of vertex pairs among the four vertices shown in Fig. 4A. As expected, the vertex pair of {1,5} versus {3,7} and that of {1,7} versus {3,5} were linearly separable along the x and z axes, respectively, with accuracies greater than 75%. Critically, the vertex pair of {1,3} versus {5,7} , a standard planar XOR problem, was also linearly separable along the v axis with accuracy greater than 75%. That is, the v axis, theoretically derived from the twist operation, was indeed present in the high-dimensional neural space (Fig. 4B).

To examine whether this twist operation can generalize from the planar XOR problem to address the cubic XOR problem (i.e., {1,2,3,4} versus {5,6,7,8}; Fig. 4C, left), in addition to one twist operation equivalent to V=Z⊕X (Fig. 4C, middle), we incorporate a second twist of the manifold’s y-v projection around the y axis to make the vertex pair of {1, 2, 3, 4} versus {5,6,7,8} linearly separable (Fig. 3C, right). In this second twist, a new axis, denoted as p=vy=zxy , is constructed. Logically, this axis is equivalent to P=V⊕Y=Z⊕X⊕Y , meaning the true and false values of P is obtained by concatenating Z , X , and Y through two XOR operators.

According to our double-twist model, successfully solving the cubic XOR classification problem would indicate a theoretical prediction, which is the emergence of an intermediate axis, referred to as the *v* axis (Fig. 4C, middle). To empirically test this prediction, we assessed linear separability of a specific classification problem of distinguishing neural responses to vertices {1,3,6,8} versus {2,4,5,7} , which should become linear separable if axis *v* really exists in neural space. The linear separability revealed by this independent analysis thus serves as empirical validation of the theoretically predicted intermediate axis *v*. The SVM analysis showed that these two sets of vertices were indeed linearly separable (Fig. 4D, middle). This finding confirms the existence of the v axis in the high-dimensional neural space. In addition, the theoretically derived p axis through double twist operations on the given sensory manifold is indeed the LR axis, as the p axis was approximately parallel to the LR axis (fig. S13). Last, the theoretically derived manifolds from twist operations (Fig. 4C) closely matched the neural manifolds derived from actual neural states (Fig. 4D) [all *R*^2^ (coefficient of determination) ≥ 0.8; for details, see Materials and Methods]. Note that the empirical v and p axes, as well as the z , x , and y axes, were mutually orthogonal, as prescribed by twist operations (Fig. 4E). This suggests that the perceptual manifold observed in the macaques’ V2 may undergo geometric transformations equivalent to the twist operations from the sensory manifold.

Evidence supporting this conjecture comes from the analysis on the latency of the emergence of the intermediate v axis, which should emerge after the sensory axes of z and x , upon which the first twist operation acts, and before the task-relevant p axis, which relies on the v axis for the second twist. Consistent with this prediction, the empirical v axis emerged (i.e., consistently exceeded the baseline) at 65 ms poststimulus onset, later than the emergence of the empirical z , x , and y axes (at 43, 57, and 53 ms, respectively), and yet earlier than that of the empirical p axis (at 87 ms) (Fig. 4F; for the time courses of all intermediate axes, see fig. S14, C and D).

Together, the presence of the intermediate v axis predicted by twist operations, not by the classification task itself, suggests that the perceptual manifold is the product of mentally processing sensory data with the involvement of NMS neurons. Next, we examined the neural geometry of the perceptual manifold and its functionality.

### Dimensionality of perceptual manifold

Given the commutative nature of the equivalent logical computation, that is P=Z⊕X⊕Y=X⊕Y⊕Z=Y⊕Z⊕X , we predicted the existence of two additional intermediate axes of u and w , which correspond to U=X⊕Y and W=Y⊕Z , respectively. Specifically, the u axis can differentiate vertices {1,4,5,8} from vertices {2,3,6,7} , and the w axis can differentiate vertices {1,2,7,8} from vertices {3,4,5,6} (fig. S12, A and C). Consistent with this prediction, the SVM analysis showed significantly higher classification accuracies for these vertex sets compared to the baseline, confirming the existence of these two intermediates u and w axes in the high-dimensional neural space (fig. S14A). Moreover, the u and w axes were orthogonal to each other and to other axes (fig. S14B).

Consequently, we identified seven mutually orthogonal axes, which can be grouped into three categories based on the number of twist operations required to derive them: the 0-twist axes (sensory axis: x , y , and z ), the 1-twist axes (intermediate axes: u , v , and w ), and the 2-twist axis (perceptual axis: p ). According to our theoretical predictions, the emergence latencies should follow the sequence: the 0-twist axes first, then the 1-twist axes, and, lastly, the 2-twist axis. To verify this prediction, we performed a bootstrap analysis (100 iterations) to calculate the emergence latency of each axis with the 95% confidence interval (Fig. 4G, left). The mean emergence latency of the 1-twist axes was 65.4 ms (SD: 8.7 ms) significantly later than the 0-twist axes (mean: 50.9 ms, SD: 6.0 ms, bootstrapped *t* testP<0.001 ) but significantly earlier than the 2-twist axis (mean: 87.0 ms, SD: 3.8 ms, bootstrapped *t* test P<0.001 ). The latency differences were statistically significant and robust, with the 95% confidence interval for the 1-twist minus 0-twist difference at [13.8 ms, 16.2 ms] and the 2-twist minus 1-twist difference at [19.0 ms, 21.6 ms], both excluding zero (Fig. 4G, right). These results confirm that the geometric transformations occur in distinct and sequential temporal stages, consistent with the predictions of the double-twist model. This pattern was replicated in the analysis of the emergence latencies of individual axes (fig. S14, C and D) and in the data of single monkeys (fig. S15).

In addition, these seven axes were present in each of three cytochrome oxidase stripes (i.e., thin, thick, and pale) in the V2 area, suggesting that the twist operation is likely a general property of V2 neurons (fig. S16). Together, the dimensionality of the perceptual manifold was at least 7, much higher than that of the sensory manifold (i.e., 3).

**Fig. 5.**
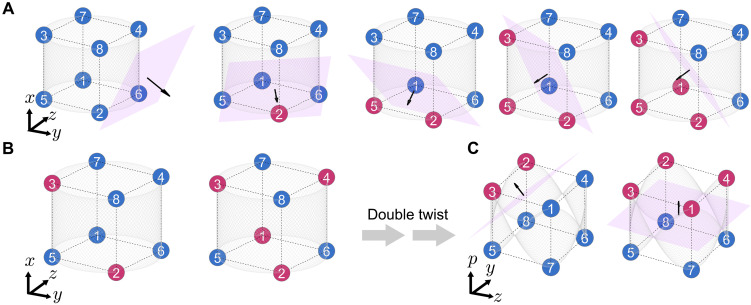
Linearly separable and inseparable classifications in the stimulus space. (**A**) Examples of linearly separable but non-axial classifications in the stimulus space. The classification separates a subset of vertices (with the set size of 0, 1, 2, 3, and 4, red) from the rest (blue). (**B**) Examples of linearly inseparable classifications in the stimulus space, including the classification of contour orientations (right). (**C**) Classifications in (B) become linearly separable in new subspaces through twist operations.

An intriguing question arises: Why was the perceptual manifold embedded in a 7D space when a 4D space, constructed by x , y , z , and p axes, is sufficient to satisfy the task demand of classifying contour orientations? One possibility is that the availability of multiple alternative pathways to construct the task-relevant p axis enhances the robustness for the classification. Alternatively, the perceptual manifold may not be task specific; rather, the classification of contour orientations could be just one of its many possible applications. For the stimulus space with eight vertices, there are $2^{8}=256$ possible classifications. Some are linearly separable in the stimulus space, such as vertex {2} versus {1,3,4,5,6,7,8} or vertices {2,5} versus {1,3,4,6,7,8} (Fig. 5A), while others are not, such as {2,3} versus {1,4,5,6,7,8} (Fig. 5B). In total, in the stimulus space, 104 classifications are linearly separable, and 152 are not (for a full list, see fig. S17 and table S1). Notably, all linearly inseparable classifications in the stimulus space become linearly separable in the 7D space. For example, the vertex pair of {2,3} versus {1,4,5,6,7,8} becomes linearly separable in the y-z-p subspace (Fig. 5C). In total, there are $C_{3}^{7}=35$ 3D subspaces embedded in the 7D perceptual space (fig. S18), and each of the 152 linearly inseparable classifications becomes linearly separable in at least one of these 35 subspaces (table S2). That is, every possible classification in the stimulus space is linearly separable in this 7D perceptual manifold.

This indicates that the perceptual manifold may not directly correspond to decision-making or action; rather, it may provide a reservoir of all possible candidates (in our case, solutions for all possible classifications) for higher-order cognitive processes. Consistent with this conjecture, after excluding task-relevant neurons that showed high sensitivity to contour orientations from the V2 neuron population, the remaining neurons retained the ability to classify contour orientations at the population level (fig. S7).

### The necessity and sufficiency of NMS neurons in dimension expansion

**Fig. 6.**
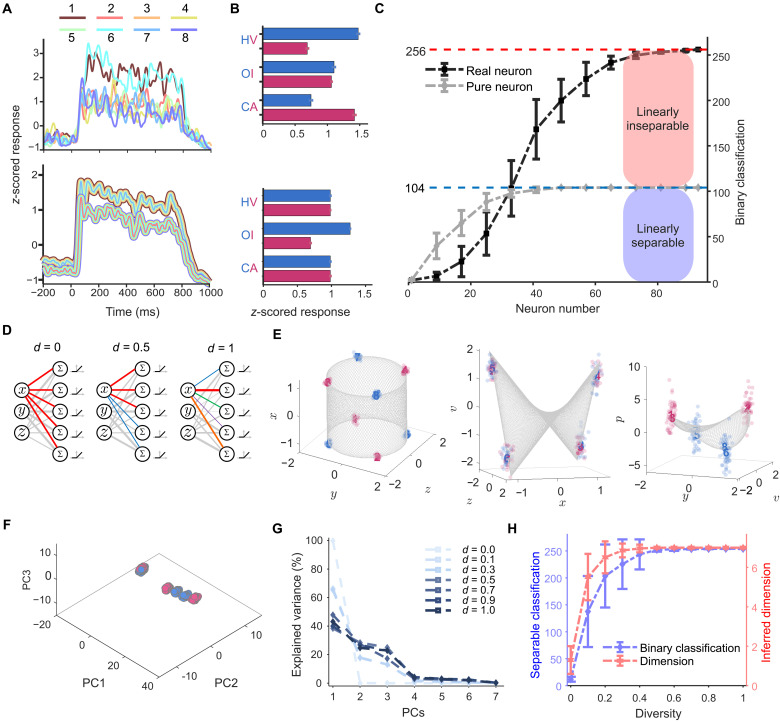
Necessity of NMS neurons and heterogeneous connectivity structure. (**A**) Time courses of an NMS neuron (top) and a synthetic neuron with pure selectivity (bottom). Colors and numbers denote different stimuli and their associated neural responses. (**B**) Average activity of the NMS neuron (top) and the synthetic neuron (bottom) from 200 to 750 ms after stimulus onset. Colors denote different conditions of a feature, and error bars denote the SE. (**C**) The number of successful linear classifications increased as a function of neuron population size for both pure selectivity neurons (gray) and NMS neurons (dark). Real neuron: neurons recorded in the V2. Pure neuron: synthetic neuron with pure selectivity. Error bar: SD. (**D**) Schematic illustration of a two-layer feedforward neural network. Letters x , y , and z represent stimulus features HV, OI, and CA. Line thickness indicates magnitudes of weights. Line colors highlight weight distribution from one neuron. Letter d : levels of diversities. (**E**) Visualization of neural manifolds when d = 1. Left: Sensory manifold that corresponds to the stimulus space. Middle: Intermediate manifold after one twist operation. Right: The subspace where linear classification of contour orientations is achieved. Red and blue represent the two contour orientations. (**F**) Visualization of the neural manifold when d = 0. The subspace is constructed by the first three PCs of neurons’ activation. (**G**) Variance explained by PCs of neurons’ activation with different levels of diversity. (**H**) Dimensionality and number of successful classifications as a function of heterogeneity in response profiles for each diversity level d . Error bar: SD.

The aforementioned analyses showed the important role of NMS neurons in expanding dimensions of representational spaces through twist operations. Here, we further examined the necessity and sufficiency of NMS neurons in dimension expansion. To do this, we first compared NMS neurons with pure selectivity neurons in carrying out all possible classifications in the stimulus space. We generated synthetic neurons exclusively tuned to one of the three stimulus features (fig. S21) based on real recorded data in V2 to simulate neurons with pure selectivity (*36*). For example, Fig. 6 (A and B) shows a typical NMS neuron in the V2 area responding differently to HV and CA and showing no sensitivity to OI (top) and a typical synthetic neuron with pure selectivity to OI (bottom).

To evaluate the classification performance for 152 linearly inseparable problems (table S1) and 104 linearly separable problems (table S2), we used a population-increment procedure (*36*), where the population size was progressively increased from a single neuron to the entire set of 93 neurons, adding one randomly selected neuron each time. During each iteration, we trained SVMs with neural activities for all possible classifications, and a classification accuracy threshold of 75% was set as the criterion for successful classification (for details, see Materials and Methods). Figure 6C shows the number of successful linear classifications as a function of neuron population size. With NMS neurons, we succeeded in all possible classifications (256 in total), for both linearly separable and inseparable problems (Fig. 6C, black curve), when the number of NMS neurons exceeded 81. In contrast, using the synthetic neurons with pure selectivity, the total number of successful classifications plateaued at 104 (Fig. 6C, gray curve) when the population size exceeded 49. That is, additional increase in neuron population size did not further improve classification performance.

Moreover, the problems successfully classified by pure selectivity neurons were all linearly separable in the stimulus space (table S1), and none came from the set of linear inseparable problems (table S2). That is, neurons with pure selectivity can only address linearly separable problems, as they alone cannot expand the sensory manifold to a higher dimensionality. Together, this finding suggests that regardless of neuron population size, NMS neurons are necessary in expanding the dimensionality of neural manifolds, hereby transforming the sensory manifold into the perceptual manifold.

The finding that at least 81 neurons were needed for forming the 7D perceptual manifold, as shown in Fig. 6C, highlights the importance of population-level activity in dimension expansion. Previous studies have shown that neurons’ diverse response play an important role in computational capacity (*44*–*46*). To quantify how diversity in response profiles of NMS neurons influences dimension expansion of the representational space, we built a two-layer feedforward neural network tasked with processing the stimuli used in the macaques’ experiment (for details on the network, see Materials and Methods). In this network, each output neuron receives the combination of all three stimulus features (i.e., HV, OI, and CA) from the input neurons and uses a nonlinear activation function (i.e., ReLU), with connectivity weights independently sampled from a multivariate Gaussian distribution. As a result, all output neurons in this neural network demonstrate a response profile of NMS.

In this network, the response profiles of NMS neurons are controlled by a parameter d , which denotes the degree of diversity in connection patterns between the two layers (Fig. 6D). This diversity ranges from identical patterns ( d=0 ) to completely uncorrelated patterns ( d=1 ) (see Materials and Methods). Whend=1 , each NMS neuron generates a distinct response because the connection pattern from the input neurons is unique (Fig. 6D, right), and therefore, the matrix of connectivity weights is full rank. SVM analysis, similar to that performed on the macaques’ data, was carried out to identify the 7D perceptual manifold. For visualization, neurons’ activations are projected into 3D subspaces (Fig. 6E), where each dot denotes the neural state of a stimulus, with red and blue colors representing the two contour orientations, respectively. Within this 7D perceptual manifold, we can identify the sensory manifold embedded in a 3D subspace constructed by axes corresponding to the three stimulus features (Fig. 6E, left), the intermediate manifold in a 3D subspace with a new axis v after one twist operation (Fig. 6E, middle), and the subspace achieved after the second twist operation where linearly separating contour orientations becomes possible (Fig. 6E, right). In addition, continuous stimuli that spanned the entire sheared configuration ring (Fig. 1C, right) produced similar results (fig. S22). In summary, the 7D perceptual manifold constructed by the network of NMS neurons with random connectivity patterns (i.e., d=1 ) is comparable to the 7D perceptual manifold identified in the macaque’s V2 (Fig. 4D).

In contrast, when d=0 , all NMS neurons have the same inputs and thus generate identical responses. Accordingly, the matrix of connectivity weights in the network is rank 1 (or 0 if all weights are 0), resulting in low dimensionality of the neural manifold (inferred dimension = 1.3, SD = 0.70, see Materials and Methods). This low dimensionality was also revealed by PCAof the variance in neuron activation (Fig. 6G). When d=0 , the first PC explained 99.84% of the total variance, leaving nearly no variance for the remaining PCs. As a result, the neural states of the stimuli were confined to an approximately 1D space (Fig. 6F). Therefore, the neural manifold constructed by the network with no diversity (i.e., d=0 ) shows substantial limitations in performing either linear or nonlinear classification (the number of linearly separable problems successfully addressed: 9.16 or 8.8% of the whole set, SD = 2.89; the number of linearly inseparable problems successfully addressed: 2.74 or 1.8%, SD = 2.23). In contrast, when d=1 , the first six PCs (99.78%) were required to explain the same amount of variance as the first PC when d=0 . Thus, when d=1 , the neural states of the stimuli were dispersed into a higher dimensional neural space. These findings suggest that networks consisting of NMS neurons with an identical response profile have limited computational capacity and thus hardly encode sufficient information, even when the response profile exhibits NMS.

To systematically investigate how diversity in the response profiles of NMS neurons influenced the dimensionality of representational spaces, we constructed a series of neural networks with different parameters d and then measured the dimensionality and the classification performances (see Materials and Methods). We found that as the diversity in response profiles increased, the dimensionality increased monotonically (Fig. 6H, red curve). The network did not need to have complete diversity to form the perceptual manifold. With d>0.5 , the dimensionality reliably expanded to 7 (see Materials and Methods and text S16). In parallel, the number of successful classifications increased monotonically, finally capable of successfully carrying out all possible classifications (i.e., 256) once the dimensionality reliably reached 7 (Fig. 6H, blue curve). Note that the linearly inseparable problems were resolved in parallel with the linearly separable ones (fig. S23).

On the other hand, heterogeneity in response profiles alone seems insufficient, as neural networks consisting of pure selectivity neurons with identical parameter d were only capable of addressing linearly separable problems (fig. S24). Together, the synergy between twist operations on input feature vectors by NMS neurons and the heterogeneous response profiles among NMS neurons is critical, which optimally leverages neural networks to construct a more complex, higher-dimensional neural space.

## DISCUSSION

In this study, we investigated how macaque V2 neurons solve linearly inseparable problems encountered in the physical world through the lens of neural geometry. By analyzing the neural geometry embedded in the high-dimensional neural space formed by collective V2 neuronal activities, we identified two related but distinct neural manifolds: the sensory and perceptual manifolds. The sensory manifold, embedded in a 3D subspace defined by the stimulus features, faithfully reflected raw sensory input, where contour orientations remained linearly inseparable. However, through a sequence of geometric transformations equivalent to twist operations, this 3D sensory manifold expanded into a 7D perceptual manifold, adding four additional axes that enabled linear separability of contour orientations. Furthermore, this dimensional expansion was achieved through the combined action of twist operations performed on input feature vectors by NMS neurons at the individual neuron level and the diverse, heterogeneous response profiles of NMS neurons at the population level. In summary, our findings offer mechanistic insight into how biological neural networks dynamically expand representational dimensionality, elucidating the transition from sensory to perceptual manifolds and thus advancing our understanding of how information progresses from sensation to perception.

Previous studies on neural geometry have shown that neural manifolds can faithfully represent both stimulus (*13**,* *18**,* *47*) and action spaces (*20*, *21*, *48*). In line with these findings, our study identified a neural manifold embedded in a 3D subspace defined by three mutually orthogonal axes corresponding to the HV, OI, and CA features of the MIC stimuli. Along with the finding that the size of this manifold was found to correlate with the intensity of motion coherence, this manifold reflects the raw sensory input (i.e., the stimulus space) and is therefore termed the sensory manifold. Although flicker features from discontinuity motion may slightly contribute to perception [see (*25*), but also see (*26*, *32*)], this study did not focus on it. Our theoretical predictions, derived from the geometric transformation of input feature vectors by NMS neurons, led to the identification of four additional axes that encode features absent from physical stimuli. Specifically, one of these axes, resulting from double twist operations on the three feature axes of the sensory manifold, encoded the perceived orientations of illusory contours, allowing for linear separability of contour orientations that were not linearly separable in the sensory manifold. This manifold was not specific to the task at hand, as it potentially accomplishes all 256 possible classifications present in the stimulus space. This manifold likely functions as an intermediary between the sensory manifold and those associated with decision-making or action, hence its designation as the perceptual manifold. Note that the perceptual manifold observed in the V2 does not necessarily originate and terminate within the V2. It likely inherits characteristics by feedforward from the V1 and is further shaped by feedback from downstream cortical regions such as the V3 and V4. Future research using simultaneous recordings across multiple areas would be valuable to elucidate the transformation from the sensory to the perceptual manifold.

The creation of these perception-related axes was attributed to NMS neurons, which have the unique ability to transform geometrically input feature vectors. The definition of mixed selectivity can, in principle, be dependent on how stimulus features and their associated axes are initially defined. However, the concept of NMS used in our study specifically denotes neuronal responses that exhibit nonlinear interactions among defined stimulus features, rather than responses that could be transformed or redefined by a linear rotation of axes. These nonlinear interacting neurons, as identified by significant higher-order interactions (e.g., via ANOVA), provide a critical computational advantage, which enables neural systems to represent and solve linearly inseparable tasks. This transformation is equivalent to twist operations, expanding the dimensions of representational spaces (*5*, *36*). In contrast, neurons exclusively selective for a single feature or those exhibiting a linear combination of selectivity for multiple features are unable to change dimensionality. Therefore, NMS neurons appear to be necessary for interpreting sensory inputs into perceptual experiences by generating latent variables from intermediate axes without direct semantic descriptions (*49*, *50*). However, the mere presence of NMS neurons is not sufficient; their functional efficacy depends on the heterogeneity of their response profiles at the population level (*45*, *51*). Through simulations of neural networks consisting of NMS neurons, we found that homogeneous response profiles among NMS neurons limited their capacity of expanding dimensions, thereby constraining the network’s computational power in addressing both linearly separable and inseparable problems. Conversely, increasing the heterogeneity of the response profiles enhanced dimension expansion, effectively transforming linearly inseparable problems into linearly separable ones. In summary, our study reveals the symbiotic relationship between the geometric transformation capability of individual NMS neurons and the heterogeneity in the response profiles at the population level, underscoring the importance of both individual neuron properties and population dynamics in achieving dimension expansion.

The high dimensionality of the perceptual manifold functions as a reservoir of computational solutions, enabling flexible classifications according to downstream task demands. That is, one key function of dimension expansion is apparently to facilitate parallel processing, allowing multiple computations (such as classifications in this study) to occur simultaneously across different dimensions. As these computations are distributed across multiple dimensions in parallel, they become less reliant on conscious control and more automatic. However, this parallel processing comes with a cost, as the number of potential solutions increases exponentially with the number of dimensions. In our study, downstream cortical regions involved in decision-making, which is usually sequential processing, must select the appropriate classification from 256 possibilities to meet the task demands. While the mechanism for effectively navigating these potential solutions based on task demands remains largely unknown, the modulation of NMS neurons’ response profiles through Hebbian (“fire together wire together”) and anti-Hebbian (“out of sync, lose the link”) rules (*52*) might offer insights into reducing dimensionality and thus narrowing the range of potential solutions. In networks governed by Hebbian plasticity, neurons frequently coactivated by similar tasks or stimuli develop more homogeneous response profiles, leading to the formation of specialized modules with relative lower dimensionality (*53*–*55*). This idea is supported by recent findings showing that sequences of contextually related images (e.g., natural video) are represented in a neural space with lower dimensionality, evidenced by straighter neural population trajectories, compared to sequences of contextually unrelated stimuli (*56*).

Conversely, under anti-Hebbian rule, task-relevant features are disentangled from task-irrelevant ones, which may result in the compression of axes representing these task-irrelevant features, thereby reducing effective dimensionality (*57*–*59*). This type of geometric transformation reshapes the representational space to focus on task-relevant features (*60*). This conjecture is supported by our findings that although the perceptual manifold can accomplish all 256 classifications, the accuracy along some dimensions (such as CA axis) was lower than others, suggesting that not all features are equally represented in the neural space. Our findings highlight that the dimensionality of perceptual neural manifolds, such as the 7D manifold identified here, inherently depends on specific task demands, stimulus sets, and learning history. Although the precise dimensionality we describe here (7D) is likely task and stimulus specific, we propose that the general computational strategy, the expansion of neural representation dimensionality through geometric twist operations via NMS, represents a flexible neural coding mechanism that is broadly applicable across diverse tasks and brain regions. Future research using learning paradigms and novel stimuli will be crucial to directly test how representational dimensionality dynamically adapts over training, how rapidly the brain can adjust its dimensional structure in response to novel computational demands, and how generalizable these neural geometric principles are across tasks and cortical areas. Together, along the hierarchy of the ventral visual stream, the amount of information encoded in each dimension varies (*61*), and the relational structure between representations evolves from relatively simple and straightforward to more abstract and complex, reflecting the integration of multiple features and the emergence of high-level perceptual categories (*61**,* *62*). The observed drop in classifier performance during the blank interval after stimulus offset suggests that the sensory and perceptual information alone may not sufficiently support the full decision-making process needed for action execution. Instead, it is likely that after initial sensory processing and perceptual formation within the V2, this information is subsequently transferred and maintained by downstream regions specialized in working memory, decision-making, and motor preparation, such as higher-order visual, parietal, and prefrontal cortices. Future studies incorporating longer intervals between stimulus offset and response initiation (*63*, *64*) and potentially using simultaneous multiarea recordings would be particularly beneficial in clarifying the respective roles and temporal dynamics of the V2 and other cortical regions during memory and decision-making processes. This intensive interplay of multiple cortical regions likely orchestrates the progression from sensory to perceptual and to decision-making manifolds and ultimately to action-oriented manifolds, which enables us to act upon the physical world in response to stimuli that has acted upon us.

## MATERIALS AND METHODS

Four hemispheres from two adult male macaque monkeys (*Macaca mulatta*) were used in this study. All procedures were performed in accordance with the National Institutes of Health Guidelines and were approved by the Institutional Animal Care and Use Committee of the Beijing Normal University [protocol number: IACUC(BNU)-NKCNL2013-13].

### Visual stimuli, behavioral tasks, and recordings

The data used in this study was from our previous work, and for more details on stimuli, tasks, and recordings, see (*32*).

Stimuli were generated with ViSaGe using MATLAB scripts and presented on a 21-inch cathode ray tube (CRT) display. MIC stimuli had seven levels of dot motion coherence. The size of the MIC was a 4° diameter circular, and the position of MIC was consistent with array population receptive field (RF).

Two monkeys performed an MIC orientation-discrimination task after headpost task training and optical imaging guided 32-channel Utah array implant. This task was a two-alternative forced-choice discrimination task, and the monkeys were trained to make an eye saccade choice based on MIC orientations. The monkeys made a saccade to the right target if the orientation was tilted to the right of the vertical axis and vice versa. The monkeys received a water reward for correct choices.

The electrophysiological recording system is AlphaLab SnR 64-channel system. Neural signals were sampled at 22 kHz and with an 800- to 7500-Hz band-pass filter. Recordings were performed on multiple days. In this study, we only used single neurons in the unique-unit dataset (*32*). This dataset was generated by excluding potential duplicated units (i.e., similar waveforms or tunings) that were recorded from the same electrodes on different days. Therefore, the neurons in this dataset were either from different electrodes or from the same electrode but had different waveforms or tunings. In addition, we further refined our selection to include only single neurons from this dataset.

### Data analysis

#### Data preprocessing

For all single neurons selected as previously described, they passed the RF test. Briefly, we used two types of RF mapping stimuli. One is grid-like RF mapping, where a 0.8° square wave grating is presented at different positions on the grid. We fit the neuronal response in two dimensions using a 2D Gaussian function. The other is 4° long and 0.2° wide bars presented at different horizontal and vertical positions, for which we use a 1D Gaussian for fitting. A goodness of fit greater than 0.7 is considered as passing the RF test [see (*32*)]. Then, to build a high-dimensional neural space, we identified all V2 neurons that participated in the MIC orientation-discrimination task. In total, we obtained 93 V2 single neurons, with 47 neurons from Monkey S and 46 neurons from Monkey W.

We sorted all trials into 112 conditions to analyze each neuron’s response (two motion-axis orientation conditions, four sheared configuration conditions, seven coherence levels, and two performance outcomes). Subsequently, we calculated each neuron’s trial-averaged response (from −200 ms before stimulus onset to 200 ms after stimulus offset) with a Gaussian window (a 10-ms sliding window with a 2-ms step size). We then combined all neuron responses after *z* -scoring each neuron’s trial average response (*15*, *65*). In addition, we excluded conditions with fewer than three trials for some neurons, so we totally got 61 useful conditions. Following these steps, we constructed a data matrix of dimensions 61 (useful conditions) × *T* (trial time) × 93 (neuron number) from the MIC orientation discrimination task.

#### Support vector machine

We used SVM for two purposes: first, to decode categorical information from the neural data, and second, to provide a well-defined vector, which represents a distinct dimension in the neural space. In the analysis, we used the *fitcsvm* function with a linear kernel in MATLAB. We retained the default hyperparameter values of the function, except for customizing the box constraint. In the neural geometry part, the box constraint was set at 0.001, whereas for the binary classification part, it was set at 1 (for an explanation of box constraints, see text S12). Then, the SVMs were trained and tested using data points in the neural space.

Since our population data are composed of multiple sessions with varying trial numbers, we followed previous methods (*15*, *65*) and used trial-averaged data (as described in the “Data preprocessing” section). So here, we divided the data at different time points into training and testing sets. First, we partitioned the complete time range from −200 to 1000 ms relative to stimulus onset into tiled 12-ms-wide time bins. Within each time bin, we randomly selected half of the time steps for training and the other half for testing. To train a single SVM classifier, the selected training data from time bins were pooled together. To generate time courses of classifier accuracy, the classifier was tested within each time bin using the testing data. We also tried another method where, instead of using tiled 12-ms time window, we randomly selected half of the time points from the entire time span as training data and the other half as test data. The classification accuracies were consistent. However, in this way, we could not obtain time courses of classification accuracy, so we did not use it in this text.

#### Angle analysis

To calculate the angle subtended between two n-dimensional unit vectors $\beta_{i}$ and $\beta_{j}$ , we used the following formula

$$\theta =\text{acos}\left(\beta_{i}^{T}\beta_{j}\right)$$

The vectors were from SVM classifications. For the neural geometry and twist model parts, we performed SVM analysis once for each classification. For control analyses, we ran SVM analysis 200 times for each classification (see text S5 and fig. S6, A and B). The orthogonality of the angle was tested by examining whether it significantly differed from the angle distribution constructed by randomly selecting two vectors in a 93D space (see text S5 and fig. S6C).

#### Low-pass filter

To smooth the temporal profiles of the neuron activities, we applied customized simple discrete-time RC low-pass filters. Let the temporal profile of a neuron be x(t) . The filtering is applied using a sliding window starting from N time steps before the current moment. In this window, the filtered temporal profile $\bar{x}\left(t\right)$ is

$$\bar{x}\left(t\right)=\alpha \;x\left(t\right)+\left(1-\alpha \right)\;\bar{x}\left(t-1\right)$$

where α is the smoothing factor. The factor α is computed from the sampling time interval Δt and the required cutoff frequency $f_{c}$ as

$$\alpha =\frac{2\pi \;\Delta t\;f_{c}\;}{2\pi \;\Delta t\;f_{c}+1}$$

Essentially, the low-pass filter computes the exponentially weighted moving average of the original temporal profile. In our study, Δt=0.002 s, $f_{c}=2$ Hz and N=5.

#### 3D visualization

The main axes identified by SVM must be perfectly mutually orthogonal for creating Cartesian coordinate systems. So here, we first created an arbitrary full rank matrix A . Its first few columns were replaced by the identified main axes by SVM. We then applied QR decomposition on matrix A to obtain an orthogonal matrix Q . The transformation matrix was $T=Q^{T}$ (see text S13 for details). Then, we used this transformation matrix to linearly transform the original neural space to a new coordinate system where the identified main axes by SVM were the first several axes.

To neatly visualize the neural states in a 3D subspace, we first applied low-pass filter to the data in the original neural space. We chose cutoff frequency $f_{c}=2$ Hz because we wanted to smooth the curve for better visualization and clearer depiction of the dynamic process. Low-pass filtering was not used in other quantitative calculations. We then used matrix T to transform the filtered data into a high-dimensional coordinate system. Last, the transformed data were projected into a 3D subspace constructed by the main axes identified for visualization.

#### Double-twist model

The double-twist model transformed the continuous cylindrical stimulus manifold depicted in Fig. 4C (left) to a continuous perceptual manifold embedded in a 7D space. Its projections into 3D subspaces were shown in gray in Fig. 4C (middle and right). The perceptual manifold arose from the correspondence between the XOR operator and the arithmetic product. Let the true value be represented by −1 and the false value by 1. The truth table of the XOR operator aligns with that of the arithmetic product (see fig. S25). That is,

X⊕Y⇔xy

where X and Y are Boolean variables and x and y are the coordinates on the x and y axes, respectively. Therefore, after two twist operations, the 7D coordinates of a point in the perceptual manifold corresponding to a point [x,y,z] in the stimulus manifold are [x,y,z,xy,yz,xz,xyz].

The derived perceptual manifold was fit to the neural data using affine transformation for visualization. Because the neural data contained noise, we first applied a low-pass filter (cutoff frequency of 2 Hz) and then calculated the steady-state averages (from 300 to 500 ms relative to stimulus onset) of neural activities for the eight stimuli to determine eight centers. These centers were then projected into various 3D subspaces (Fig. 4). In each subspace, we located the neural states for the eight stimuli based on the derived perceptual manifold. Using the least square method, we obtained the transformation matrix F . The mean of the residuals was represented as a vector e . For any point x on the 3D projections of the derived perceptual manifold, we applied the transformation

$$\hat{y}=Fx+e$$

to fit the model to the data (see text S14 for detailed formulation). The goodness of fit was measured by *R*^2^.

#### Binary classification

Binary classification entailed sorting stimulus conditions into two classes based on all possible classification rules. With eight stimulus conditions, we had a total of 256 classification rules. For each classification, the criterion for linear separability was set at 75% accuracy within each stimulus condition. Each neuron pool was randomly selected from the entire set of 93 neurons, one at a time, and we then conducted SVM analysis 10 times for each neuron pool (see text S15).

To infer the dimensionality from binary classifications, we used the approach developed by Rigotti *et al.* (*36*). Briefly, we calculated the ratio of the actual number of linearly separable classifications and the theoretical number of binary classifications for each condition number n ( n∈{2,…,8} ). The last n whose ratio > 0.8 was selected as the dimensionality (see text S16).

#### Connectivity patterns of neural networks

We created a simple two-layer feedforward neural network; the first layer contained three units representing the three feature dimensions, respectively, and the second layer contained 93 NMS neurons. Each neuron received the input signals of all the three feature dimensions (mixed): x(HV), y(OI), and z(CA) and used a ReLU activation function (nonlinearity). The input signals represented the eight corners of the cube in the x-y-z stimulus space, each corresponding to a specific stimulus. The activity $r_{i}\;$  of the i th neuron was defined as

$$r_{i}=\text{ReLU}\left(\sum_{j}w_{\mathrm{ij}}\gamma_{j}+b_{i}\right)+\epsilon$$

where $\gamma_{j}\in \left\{x,y,z\right\}$ is the input signal of the j th stimulus feature with $w_{\mathrm{ij}}$ as the weight, $b_{i}$ is a random bias sampled from the uniform distribution 𝒰(0,1) , and ϵ is a noise term drawn from the Gaussian distribution 𝒩(0,1/3).

The weights $w_{\mathrm{ij}}$ were randomly sampled from standard Gaussian distributions. The covariance between the weights controlled the structure of the network and thus determined the heterogeneity of the output activities. Hence, we used a diversity parameter d∈[0,1] to define the 93D × 93D covariance matrix K for sampling the weights of feature x

$$K=\left[\begin{array}{cccc} 1 & 1-d & \cdots & 1-d\\ 1-d & 1 & \cdots & 1-d\\ \vdots & \vdots & \ddots & 1-d\\ 1-d & 1-d & \cdots & 1 \end{array}\right]$$

The covariance matrix K was fed into the *multivariate_normal* function in Python’s *numpy.random* package to generate the weights. The procedure for generating the weights of features y and z was identical.

We ran multiple simulations on the network. In each simulation, input stimuli were repeated 100 times to allow the added random noise for generating point clusters. We applied the same analyses for the network’s output activities as we did for the neural data. One hundred simulations were conducted to calculate the dimensionality of the neural geometry.

## Supplementary Materials

### The PDF file includes:

Supplementary Text S1 to S16 Figs. S1 to S25 Tables S1 and S2 Legends for movies S1 and S2 References
