# Supplementary material for: From sensory to perceptual manifolds: The twist of neural geometry
Source: Sci Adv. 2025 Dec 10;11(50):eadv0431. doi: 10.1126/sciadv.adv0431 (PMC12693994; doi:10.1126/sciadv.adv0431)
Supplement: Supplementary file 1 — Supplementary Text S1 to S16 Figs. S1 to S25 Tables S1 and S2 Legends for movies S1 and S2 References [file sciadv.adv0431_sm.pdf]

Science Advances
AAAS

Supplementary Materials for

From sensory to perceptual manifolds: The twist of neural geometry

Heng Ma *et al.*

Corresponding author: Jia Liu, liujiathu@tsinghua.edu.cn

Sci. Adv. **11**, eadv0431 (2025)
DOI: 10.1126/sciadv.adv0431

The PDF file includes:

Supplementary Text S1 to S16
Figs. S1 to S25
Tables S1 and S2
Legends for movies S1 and S2
References

Other Supplementary Material for this manuscript includes the following:

Movies S1 and S2

Supplementary Text

T,1 MIC stimuli and stimuli space

The standard MIC stimuli consist of two main parameters. Fig. S1A represents the orientation of the axis of oppositely moving dots, while Fig. S1B represents the sheared angle of the neighboring dots moving in the same direction (see Fig. 1B in the Result section). Our work focused on four conditions (represented by black dots in Fig. S1B), while other works (referred to as KEG and KEO stimuli, 24,25) explored the additional conditions, essentially sampling from the sheared configuration space.

For the MIC orientation discrimination task, we utilized the standard MIC stimuli and added 7 levels of coherence to the moving dots (32). Specifically, we selected horizontal-vertical conditions (±15° compared to horizontal or vertical orientation) for the motion-axis orientation and used the four conditions mentioned earlier for the sheared angle. This resulted in a total of 8 stimuli per coherence level. By doing this, we could project these two independent parameters (each having one intrinsic dimension) into 3 dimensions in Euclidean space under Cartesian coordinates.

T.2. Sheared configurations of stimuli

To further explain what sheared configurations are, we illustrated how they determine the dot movement of the stimuli and how they were parameterized as a complete space. In the ideal stimuli, the dots move coherently in the two halves of the viewing area in opposite directions. The sheared configurations specify the relative locations of neighboring dots where the coherent movement experiences abrupt change, that is, between the two oppositely moving halves of the stimuli. In other words, it is where the moving dots appear or disappear. Fig. S2A & B depict two examples of sheared configurations for when the motion axis is horizontal. Only half of the viewing area is used in the illustration. The sheared configurations were parameterized by an angle θ with respect to the motion axis. Due to the coherent motion of the dots, the sheared configuration between any two neighboring dots remains constant through the dot presentation in

the viewing area. The two θs in Fig. S2 A & B are sampled from a circle (see color coding) in which each point represents a distinct sheared configuration for $\theta \in [0, 2\pi]$ (Fig. S2C). The circle lies in a 2-D space which can be spanned by two orthogonal axes, each as the feature of the space. The names of the feature axes derive from the stimuli patterns directly on the axes, respectively.

T.3. Topology of the entire stimulus manifold

The focus of this study enabled us to reduce the stimulus manifold to a cylindrical manifold (Fig. 1D). This is because we used only the horizontal-vertical motion axes in the stimulus set (explained in Fig. S1). However, the complete stimulus manifold is more complex; it is a Clifford torus in a 4-D space whose topology is visualized in Fig. S3 (left). The cylindrical surface is a 3-D projection of the 4-D torus (Fig. S3, right).

T.4. Population Average Response and Principal Component Analysis (PCA)

To measure the neural data's ability to differentiate four types of classifications (HV, OI, CA, and LR), we employed traditional methods, including population average response analysis and PCA.

In population average response analysis (Fig. S5A), we first z-scored the trial average response of each neuron. For one type of classification (e.g., H versus V in the HV classification), we selected higher average responses and represented them as warm color curves, while lower average responses were represented as cold color curves. By aligning all neuron responses based on higher or lower responses, the difference in response between warm and cold color curves indicated the neural data's ability to differentiate the HV conditions.

For the PCA analysis (Fig. S5B), we used the high-dimensional neural data and considered each neuron as one dimension. Through the PCA analysis (Matlab2017b toolbox), we obtained 93 orthogonal principal components (PCs). We then projected the population neural responses from the time interval of 200–700ms into this orthogonal PCs space. The PCA results revealed that the 8 conditions formed 8 clusters, and more than three PCs had significant loading weights for these

clusters. Because PCA is a linear transformation, it allowed us to transform our neural data from a neuron-based representation to a PC-based representation, where each PC was found by variance in the data. However, it is important to note that individual PCs did not have actual meanings and thus lacked direct interpretability. Our data showed that more than one PC contributed to each of the HV, OI, CA, and LR classifications, indicating that the classification directions of the classifications could not be precisely obtained through the PCA analysis. Consequently, we chose the linear SVM method to identify the classification directions for further analyses.

T.5. Angle analysis

Angle distribution.

For neural geometry in Fig. 2 and Fig. 4, we performed SVM analysis once for each of the HV, OI, CA, and LR classifications and obtained the classification directions. Accordingly, Fig. S6A shows the distribution of $\binom{4}{2} = 6$ angles of the 4 different classification directions. For control analysis, in Fig. S6 B & C, we ran SVM analysis 200 times for each classification (training and testing sets were directly sampled in the range from -200ms to 1000ms randomly) to obtain 200 directions for this classification. So, we got $\binom{4}{2} \times 200 \times 200 = 240{,}000$ angles between directions of any two different classifications and $\binom{200}{2} \times 4 = 79{,}600$ angles between directions of a same classification among the 4 different classifications. These two angle distributions are shown in Fig. S6B. The angle distribution of different classifications was significantly larger than the angle distribution of a same classification ($p < 0.001$, t-test), with mean values of 92.69 ± 1.94 degrees and 6.82 ± 1.06 degrees, respectively (mean ± standard deviation).

Because the angle distribution between two random vectors is likely more concentrating to 90 degrees as space dimension becomes higher (70), to validate whether the orthogonality observed in Fig. S6A was different from randomly selecting two vectors in high-dimensional space, we constructed an angle distribution between two random vectors in the 93-dimensional space (Fig.

S6C). For this calculation, we followed the method mentioned below (i.e., "Angle between two random high-dimensional vectors"). We found that the resulting distribution (90.02 ± 5.97 degrees, mean ± standard deviation) was significantly different ($p < 0.001$, Kolmogorov-Smirnov test) from the angle distribution of different classifications. The angle distribution of random vectors had a larger standard deviation. Additionally, this wider distribution was similar to the angle distribution obtained after shuffling data labels.

Angle between two random high-dimensional vectors.

Theoretically, when two-unit vectors are randomly directed in a n-dimensional space and n is large, the subtended angle is close to 90° (70). To demonstrate that our classification directions are indeed orthogonal with each other, we conducted a control analysis by sampling the subtended angles of two randomly directed n-dimensional vectors. We followed the procedure of sampling one randomly directed vector (71), that ensures all the sample vectors are uniformly distributed on the surface of an $(n-1)$-sphere (72). The procedure is as follows:

1. Sample the n components of a vector $\boldsymbol{\beta}_i$ independently from standard normal distribution $\mathcal{N}(0,1)$.
2. Normalize the sampled $\boldsymbol{\beta}_i$ to obtain a unit vector.

We randomly sampled 240,000 pairs of vectors, which is the same number as the angles of different classifications in Fig. S6B. For each pair, we calculated the subtended angle between the two vectors, resulting in an empirical angle distribution.

T.6. Neural contribution and population coding

To study the characteristics of neural population encoding, we analyzed neural loading weights for each dimension, HV, OI, CA, and LR, and performed SVM analysis while gradually reducing the number of neurons. The results showed that each neuron had mixed selectivity for all dimensions (Fig. S7A). For the reducing neuron analysis, after removing neurons with less mixed selectivity, the classifier accuracy did not decrease sharply; instead, the accuracy decreased gradually with smaller number of neurons (Fig. S7B).

T.7. Linearly separable classifications

The number of linearly separable classifications

The number of linearly separable classifications is 104. We obtained this number by enumeration. Fig. S17 shows how the number of linear separability was enumerated.

Enumeration of linearly separable classifications

In Table S1, we listed all possible linearly separable classifications. Since the classifications are symmetric, there are 52 unique classifications listed in the stimulus space. The number of classifications was enumerated as in Fig. S17. The SVM analysis on the synthesized pure selectivity neural data (Fig. S21) confirmed that the classifications listed in this table are indeed separable, with a minimal accuracy of 95.7% when the box constraint was set at 1.

Enumeration of linearly inseparable classifications

In Table S2, we list 9 subspaces that have already provided 152 linearly separable versions of the classifications that are linearly inseparable in the stimulus space. Since many classifications are symmetric, we list 152/2=76 unique classifications in 9 subspaces after excluding the symmetric ones. The subspaces are shown in Fig. S18. The 9 subspaces all require at least one of the u, v, w, and p axes. Linear classifications in other subspaces are replicates of the ones listed here.

T.8. Independence of classifications

Definition of independent classification

Denote a classification as grouping a set of data points into two subsets: a subset X versus the complement X^c. Two independent classifications of the same set, X versus X^c and Y versus Y^c, have the property that knowing an object a belonging to X does not affect the probability of correctly guessing whether a belongs to Y. That is, the two classifications are completely uncorrelated.

The classifications for HV, OI, and CA are indeed mutually independent (see below). Their classification directions in the neural space were mutually orthogonal. Intriguingly, the orthogonality corresponded nicely to the independence of classifications. This correspondence motivated us to define mathematically what dependent and independent classifications are. Here, we explain the conditions for two different classifications to be independent. Suppose the set U contains all the data points. Non-empty and non-overlapping subsets cover U: $A \cup B \cup C \cup D = U$. Let the first binary classification be $X = A \cup B$ versus $X^c = C \cup D$. Let the second binary classification be $Y = A \cup C$ versus $Y^c = B \cup D$. We then get $X \cap Y = A$, $X \cap Y^c = B$, $X^c \cap Y = C$, and $X^c \cap Y^c = D$. For an arbitrary data point a, we derive the condition under which $P(a \in Y | a \in X) = P(a \in Y)$. According to Bayes' rule, we derive

$$P(a \in Y | a \in X) = \frac{P(a \in Y, a \in X)}{P(a \in X)} = \frac{\|X \cap Y\|}{\|X\|} = \frac{\|A\|}{\|A \cup B\|} = \frac{\|A\|}{\|A\| + \|B\|}.$$

On the other side, we derive

$$P(a \in Y) = \frac{\|Y\|}{\|U\|} = \frac{\|A \cup C\|}{\|A \cup B \cup C \cup D\|} = \frac{\|A\| + \|C\|}{\|A\| + \|B\| + \|C\| + \|D\|}.$$

Equalizing these two expressions and simplifying, we obtain

$$\frac{\|A\|}{\|B\|} = \frac{\|C\|}{\|D\|}.$$

Furthermore, if we additionally have $\|X\| = \|X^c\|$ and $\|X\| = \|Y\|$, we can derive $\|A\| = \|B\| = \|C\| = \|D\|$. In our work, given one classification X and X^c with $\|X\| = \|X^c\| = 4$, if we swap 2 elements in X with 2 elements in X^c, we get a new classification Y and Y^c that is independent to the original one.

Properties of dependent and independent classifications

With respect to binary classifications where each class has 4 elements, there are $\binom{8}{4}/2 = 35$ different classifications, after excluding symmetric ones. As discussed above, any two classifications are either independent or dependent. Using linear SVMs, we computed the accuracies of these classifications and found classification directions of these separations. All the classification directions existed according to the classification accuracies (Fig. S20A). We

computed the angles between the classification directions of the independent classifications and the angles between the classification directions of the dependent classifications and showed their distribution in Fig. S20B.

T.9. Synthesizing purely selective neurons

Using ANOVA tests, we found all the recorded neurons had nonlinearly mixed selectivity. In order to compare classification capabilities, we created purely selective neurons. (Purely selective neurons are special cases of linearly mixed selective neurons, i.e., zero interaction effects, thus in essence they are the same.) We did this by synthesizing pure neuronal responses from the raw neural data to maximally preserve data variability (36). Fig. S21 shows the procedure of synthesizing one neuron purely selective to HV from a recorded example neuron.

T.10. Continuous manifolds can be observed in simulated network data

In the Result section, we focused on showing the neural data of the 8 stimuli and drew the manifold predicted by the double-twist operations as the background. That is, the neural data were disconnected clusters on the manifold. To further illustrate the continuity of neural manifolds suggested by the model, we used continuous stimuli as inputs to the neural network (Fig. S22) and analyzed the output data.

Specifically, the continuous stimulus input spanned the entire sheared configuration rings (Fig. S1B) for the vertical and horizontal motion-axes, respectively. The diversity parameter was set at d=1 for the neural network. Thus, the input space was two 1-D rings (Fig. S22A, left), with 400 samples uniformly distributed on each ring. The ring-shaped neural states corresponding to the input structure was evident in Fig. S22B (left).

We then fitted the theoretically-derived perceptual manifold to the data points corresponding to the 8 stimuli in each 3-D subspace, which labelled either in red or blue (Fig. S22). Each stimulus points were computed as the average of 30 data points around the solid circles in Fig. S22A (left). Affine transformation was used for the fitting. Similar manifold fittings were observed in

other subspaces. The illustration of the simulated network data and the fitted perceptual manifolds are shown in Fig. S22.

T.11. Arithmetic product as continuous version of exclusive OR (XOR)

There is correspondence between XOR and arithmetic product. Let us correspond the Boolean true value to -1.0 and the Boolean false value to 1.0 as shown in Fig. S25. For instance, a Boolean variable X is true if and only if the corresponding real variable x = -1.0, and false if and only if x = 1.0. We find that the truth values of $X \oplus Y$ align well with the results of $x \cdot y$.

Unlike XOR operation, arithmetic product can deal with continuous values, i.e., $x, y \in \mathbb{R}$. Hence, arithmetic product can be regarded as a continuous version of XOR operation. With continuous variables, arithmetic product effectively renders a twisting operation shown in Fig. 4A.

T.12. Explanation of support vector machine (SVM)

The SVM used in this study is a binary linear classifier that maximizes the margin between the cluster boundaries of two classes of data points in high-dimensional spaces. Formally, let $\boldsymbol{x}$ be a point in the n-dimensional neural space. With respect to a stimulus feature, a point $\boldsymbol{x}$ can be labelled by $y = \pm 1$, corresponding to the positive and negative classes, respectively. The class separating hyperplane is represented by the equation:

$$f(\boldsymbol{x}) = \boldsymbol{x}^{\mathrm{T}}\boldsymbol{\beta} + b = 0,$$

where $\boldsymbol{\beta}$ is the unit vector representing the direction of the hyperplane, and b is the bias along the direction from the origin of the space. The predicted class of $\boldsymbol{x}$ is determined by the sign of $f(\boldsymbol{x})$. The optimization process aims to find the optimal values of $\boldsymbol{\beta}$ and b that maximize the margin. The optimized $\boldsymbol{\beta}$ gives us the classification direction.

Specifically, after mathematical derivations (73), for all labelled data points $(\boldsymbol{x}_i, y_i)$ the optimization is

$$\min_{\boldsymbol{\beta},b,\xi}\left(\frac{1}{2}\boldsymbol{\beta}^{\mathrm{T}}\boldsymbol{\beta} + c\sum_i \xi_i^2\right),$$

such that

$$y_i f(\boldsymbol{x_i}) \geq 1 - \xi_i, \ \ \xi_i \geq 0,$$

where $\boldsymbol{\xi} = [\xi_1, \xi_2, \ldots]$ represents the slack variables that allow soft separation. The value of the box constraint c controls the softness of the separation.

Softness is introduced to prevent overfitting of the SVM classifier. A softer separation permits more classifying errors, while a harder separation allows fewer classification errors. Careful selection of the box constraint is necessary to ensure the classifier is accurate enough without being overfitted with noise.

T.13. Algorithm of linear transformation for 3-D visualization

Suppose we have m mutually orthogonal main axes which are n-dimensional unit vectors $\boldsymbol{v_1}, \boldsymbol{v_2}, \ldots, \boldsymbol{v_m}$. The algorithm to generate linear transformation matrix $\boldsymbol{T}$, from the original neural space to the new coordinate system, is in Algorithm 1.

Lines 1–2 initialize a matrix $\boldsymbol{A}$ with information of the main axes embedded. Line 3 converts $\boldsymbol{A}$ into an orthogonal matrix $\boldsymbol{Q}$ so that it can be a transformation matrix. The first m columns of A were generated by SVM classifications. Empirically, the first m columns are approximately orthogonal (16,74). Accordingly, the first m columns of $\boldsymbol{Q}$ are approximately parallel to the first m columns of $\boldsymbol{A}$. Lines 4–9 ensure the first columns of $\boldsymbol{Q}$ and $\boldsymbol{A}$ have same instead of opposite directions. The output line follows the fact that the relation between a data point's description $\boldsymbol{b}$ in the new coordinate system and the data point's description $\boldsymbol{a}$ in the original coordinate system is $\boldsymbol{a} = \boldsymbol{Qb}$. We can use $\boldsymbol{b} = \boldsymbol{Q}^{-1}\boldsymbol{a}$ to get the description in the new coordinate system. Since $\boldsymbol{Q}$ is an orthogonal matrix, we get the transformation matrix as $\boldsymbol{T} = \boldsymbol{Q}^{-1} = \boldsymbol{Q}^{\mathrm{T}}$.

Algorithm 1: **Linear transformation matrix**

Input: $\boldsymbol{v_1}, \boldsymbol{v_2}, \ldots, \boldsymbol{v_m}$

1. Initialized a $n \times n$ identity matrix $\boldsymbol{I}$
2. Replace the first m columns of $\boldsymbol{I}$ with $\boldsymbol{v_1}, \boldsymbol{v_2}, \ldots, \boldsymbol{v_m}$ to get a new matrix $\boldsymbol{A}$.
3. Use QR decomposition $[\boldsymbol{Q}, \boldsymbol{R}] = \mathrm{qr}(\boldsymbol{A})$ in Matlab to get an orthogonal matrix $\boldsymbol{Q}$ and an upper triangular matrix $\boldsymbol{R}$.

```
    For i = 1 ... m
        If the ith diagonal element of R is negative
            Change this element to positive
            Prefix a negative sign to the ith column of Q.
        End
    End
Output: T = Q^T
```

T.14. Fitting the double-twist model to neural data

We used affine transformation to fit the double-twist model to the neural data. We first applied the low-pass filter (cutoff frequency: 2Hz) to reduce the noise. Then we computed the steady-state averages (300ms–500ms relative to stimulus onset) of the neural activities for the 8 stimuli to get 8 data centroids. The 8 data centroids $\boldsymbol{y}_i$, $i = 1, \dots, 8$, in each 3-D subspace were stored as columns in a matrix $\boldsymbol{Y}$.

We found that 8 locations $\boldsymbol{x}_i$ on the ideal double-twisting model corresponded to the 8 data centroids in the same 3-D subspace. These locations were stored as columns in $\boldsymbol{X}$. We wanted to find a transformation $\boldsymbol{Y} = \boldsymbol{F}\boldsymbol{X}$. The matrix $\boldsymbol{F}$ was computed using the least-square method as $\boldsymbol{F} = \boldsymbol{Y}\boldsymbol{X}^{\mathrm{T}}(\boldsymbol{X}\boldsymbol{X}^{\mathrm{T}})^{-1}$. However, the least-square method generated nonzero residuals $\boldsymbol{E} = \boldsymbol{Y} - \boldsymbol{F}\boldsymbol{X}$. We computed the mean of the column vectors of $\boldsymbol{E}$ as the residual vector $\boldsymbol{e}$. The matrix $\boldsymbol{F}$ and the vector $\boldsymbol{e}$ constituted the affine transformation. For any point $\boldsymbol{x}$ on the ideal double-twist model, we applied the transformation:

$$\hat{\boldsymbol{y}} = \boldsymbol{F}\boldsymbol{x} + \boldsymbol{e}.$$

The goodness of fit between 8 data points $\boldsymbol{y}_i$ and 8 fitted locations $\hat{\boldsymbol{y}}_i$ was computed using R^2 as

$$R^2 = \frac{\sum_{i=1}^{8} \|\hat{\boldsymbol{y}}_i - \bar{\boldsymbol{y}}\|^2}{\sum_{i=1}^{8} \|\boldsymbol{y}_i - \bar{\boldsymbol{y}}\|^2}.$$

T.15. Binary classification

Binary classification involves classifying stimulus conditions into two classes based on all possible classification rules. In this context, the number of all possible classification rules is 2^N,

where N represents the number of stimulus conditions. As we had 8 stimulus conditions, there were $2^8 = 256$ classification rules in total. The neural states for the same stimulus condition were clustered, and thus neural space had 8 clusters in total. Accordingly, for each classification, the criterion for linear separability in the neural space was set to achieve an accuracy above 75% for each stimulus cluster, rather than considering the overall accuracy across all stimulus states. In the actual analysis, we only tested 127 classification rules because the other half of the classification rules are symmetric to them, and all conditions belonging to one category are inherently linearly separable.

In order not to underestimate the number of linearly separable classifications because of noisy stimulus, here we only used data from coherence level 7 and correct trials, where box constraint c for SVM analysis was set to 1. In addition, each neuron pool consisting of varying number of neurons was randomly selected from the whole 93 neurons one at a time, and the SVM analysis was conducted 10 times for each neuron pool. In the case of the neuron pool that already included all 93 neurons, we conducted the SVM analysis only once.

T.16. Dimensionality of artificial neural networks

We calculated the dimensionality of the given artificial neural network with a specific diversity d. Firstly, we chose a number n ($n \in \{2, \ldots, 8\}$) increasingly from 2 to 8, and randomly selected n conditions from the whole 8 conditions. Then, we performed SVM analysis on these n conditions to obtain the actual number of linearly separable classifications and calculated the theoretical number of binary classifications as 2^n. Finally, we calculated the ratio of these two numbers. In this way, we got one ratio for each n. The last n whose ratio > 0.8 was selected as the dimensionality of this network. Using this procedure, we ran 100 simulations of the networks, obtaining 100 dimensionality for each diversity d. We calculated means and standard deviations of the dimensionality for different diversity d and plot them as in Fig. 6H.

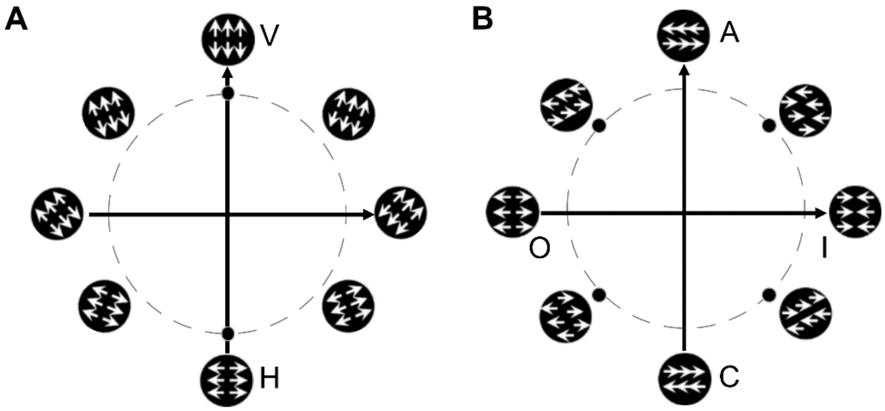

Fig. S1. Whole stimuli space. The whole stimuli space for MIC. MIC stimuli were constituted by two intrinsic dimensions. Panel A represents the orientation of axis of oppositely moving dots, and we chose 2 conditions (black dots) for further analysis. Panel B represents sheared angle of the neighboring dots moving in the same direction, and we used 4 conditions (black dots) in this sheared configuration space. In this way, we constructed three stimuli dimensions HV, OI and CA to describe the stimulus space of the discrimination task.

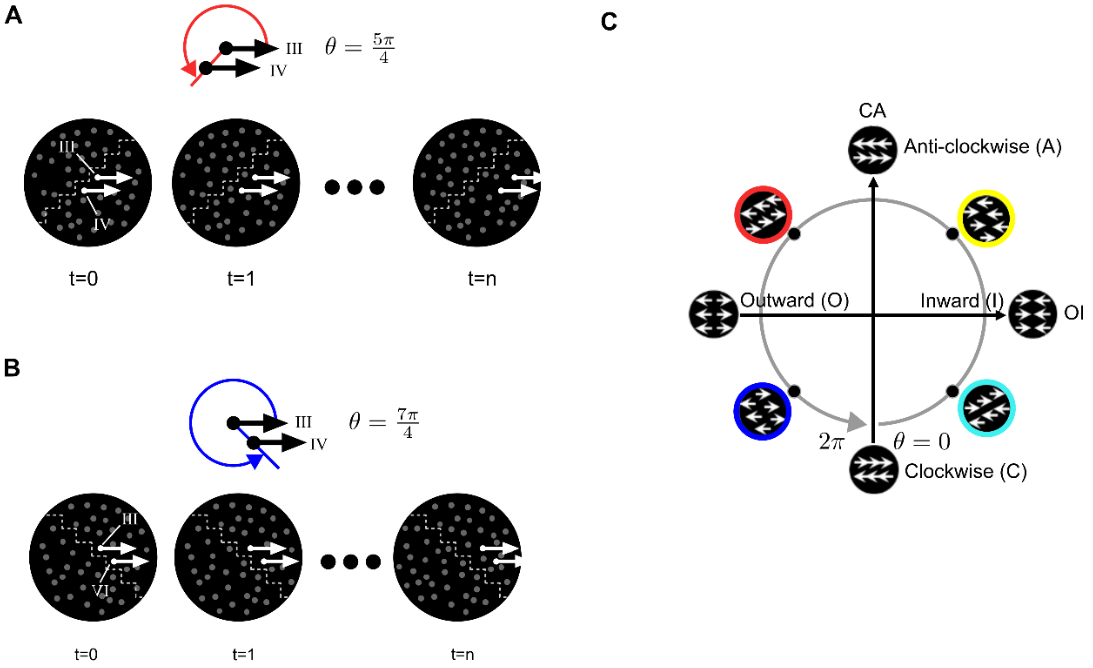

Fig. S2. Sheared configurations of stimuli. (A) An exemplar sheared configuration for neighboring moving dots ($\theta = 5\pi/4$). Because of the coherent motion of the dots, this particular sheared configuration remains constant throughout the dot presentation in the viewing area. (B) Another exemplar sheared configuration ($\theta = 7\pi/4$). (C) The whole space of the sheared configurations. These configurations collectively form a 2-D circle, with $\theta \in [0, 2\pi]$ and $\theta = 0$ positions at the bottom of the circle. Two orthogonal axes extend across the 2-D space where the circle is embedded. According to stimulus patterns on the axes, the axes are therefore named the outward-inward (OI) and clockwise-anticlockwise-like (CA) axes, respectively.

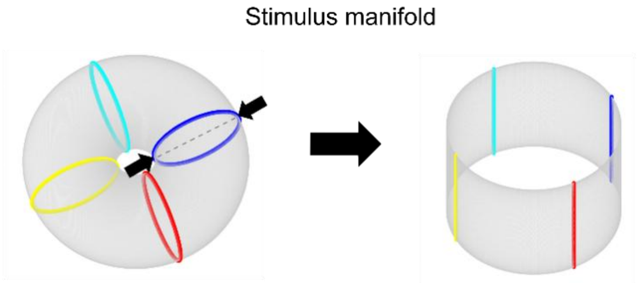

Fig. S3. Topology of stimulus manifold. The entire stimulus manifold takes the form of a Clifford torus in a 4-D space (66). Left: For visualization, the topology of the stimulus manifold is illustrated in a 3-D form, where each colored circle corresponds to a subspace of motion axes. Distinct motion axes are represented by points on these circles, with the color codes inherited from the sheared configurations depicted in Fig. S2C: Given our study's focus on just two conditions in the motion axis subspace, namely horizontal and vertical, we can project the 4-D Clifford torus along an unrelated axis (as shown by the dashed line) within the motion axis subspace. This projection transforms the torus into a cylinder embedded in a 3-D space, thereby converting the colored circles into line segments (Fig. 1D).

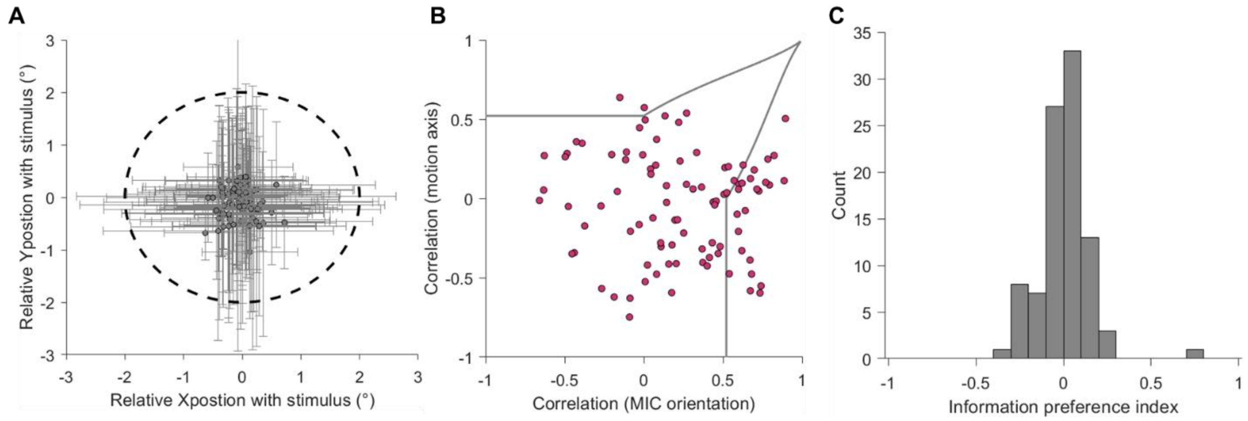

Fig. S4. Neurons' receptive fields and selectivity. (A) Spatial alignment of neurons' receptive fields (RFs, the horizontal and vertical lines represent the range of the RFs centered by a circle point for the neurons.) with the size of the stimuli delineated by a dotted circle. The size of the stimuli was slightly larger than that of neurons' RFs (2.50±0.90°). (B) Neurons' selectivity based on a partial correlation analysis (24,67,68). The data used here was from testing MIC basic tuning under passive fixation (32). Following the methods of previous researchers (24), we had two sets of MIC stimuli, each set contained 12 contour orientation conditions, differing in the angle between contour orientation and motion axis. The abscissa represents the correlation coefficient between neural responses for these two sets according to MIC orientation. The ordinate represents the correlation coefficient between neural responses for these two sets according to motion axis. Neurons closer to the top-left showed greater responsiveness to motion information (motion axis), while those nearer the bottom-right were more influenced by MIC information. (C) Neurons' selectivity using the Epoch-preference index method (69). Similar to panel B, this index was used to compare neural responses driven by MIC orientation and motion axis. Positive values indicate that a neuron is more selective for the motion axis, while negative values indicate that a neuron is more selective for MIC orientation. The distribution of this index did not significantly deviate towards bimodality (Hartigan's dip test; dip statistic = 0.023; p = 0.99), indicating that neurons were approximately equally responsive to both motion and MIC information.

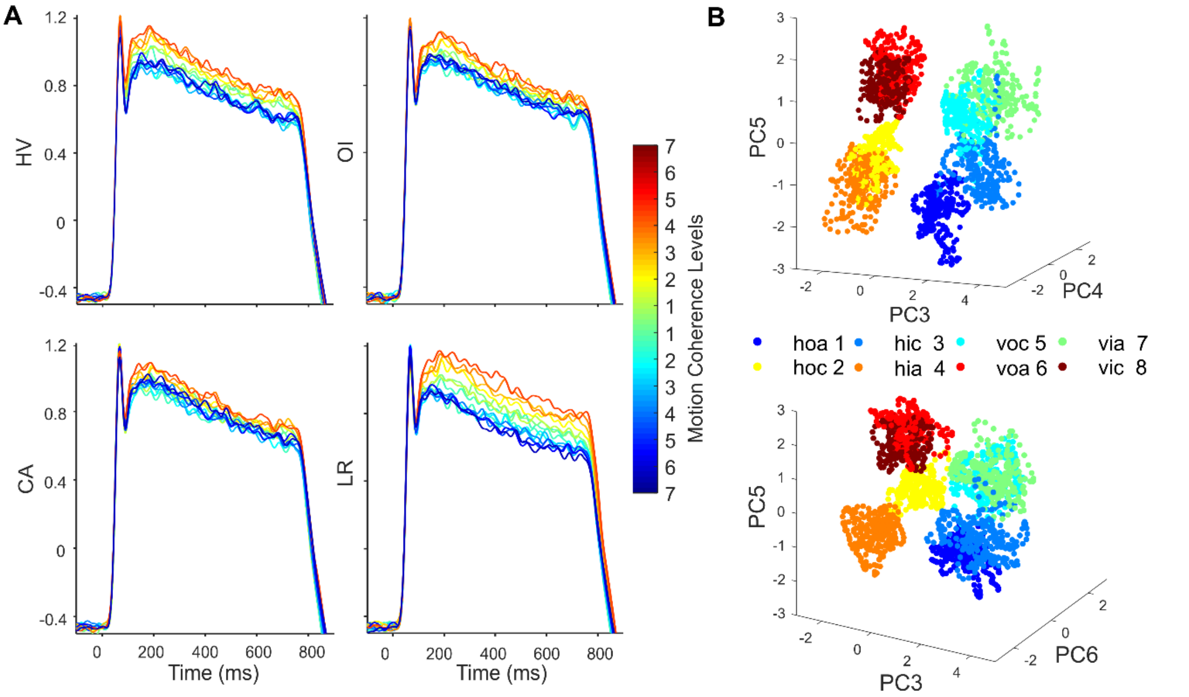

Fig. S5. Population related results. (A) Z-scored population results aligned by related preference. Four panels represent four types of classifications. Different colors represent the curves for different signal strengths, symmetrically. These panels reflect the ability to measure the difference between two conditions of a category with the traditional average response method. Colors denote levels of motion coherence. (B) PCA results. The top and bottom panels used different PCs to show 3-D projections. Eight different colors represent 8 conditions. The 8 stimulus conditions formed 8 clusters, and more than 3 PCs had significant loading weights for these clusters.

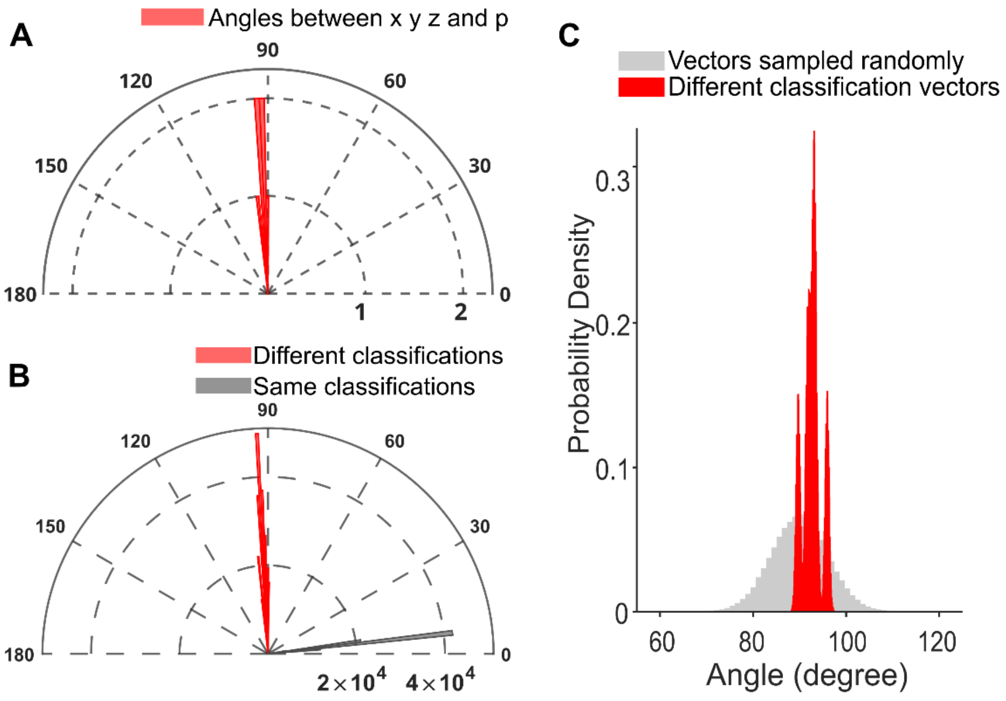

Fig. S6. The control analysis of angle distribution. (A) Angle distribution of the HV (x), OI (y), CA (z), and LR (p) classifications. Angles were calculated for each pair of vectors in the 4 classification directions. The angles concentrated tightly around 90°. (B) Angle distributions of different classifications versus angle distributions of the same classification obtained from SVM. The distribution of different classifications (red) concentrated tightly around 90°. The distribution of the same classification (grey) concentrated tightly near 0°. (C) Angle distributions of different classifications versus angle distributions of random vectors in a 93-dimensional space. The distribution of different classifications was significantly more concentrated than the distribution of random vectors.

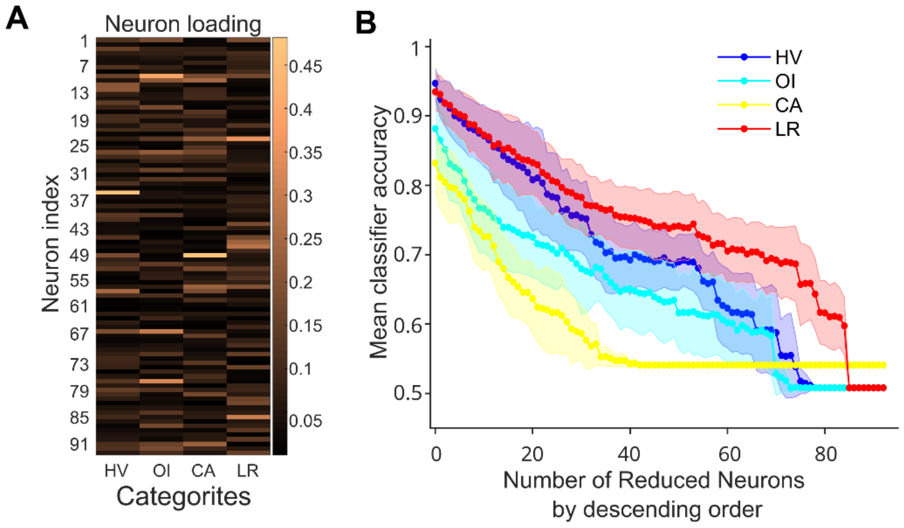

Fig. S7. Neuron loading weights and the signature of population coding. (A) The matrix of neuron loading weights for the 4 classifications: HV, OI, CA, and LR. Brown color intensity represents the absolute value of loading weights (i.e., the component of a given classification direction on each neuron). As seen, the majority of the neurons had bright color, indicating their participation in specifying the classification directions. (B) Relationship between mean classifier accuracy and the number of neurons. Neurons were removed based on the descending order of the absolute value of their loading weights for each type of classification. Curves with shaded areas represent mean accuracy ± 3 standard deviations (from 200ms to 750ms), and colors represent the 4 types of classifications. Analyses of the loading matrix and the gradual removal of neurons showed that each single neuron contributed to the classifications, and the robustness of classifier accuracy did not decrease sharply upon reducing more selective neurons.

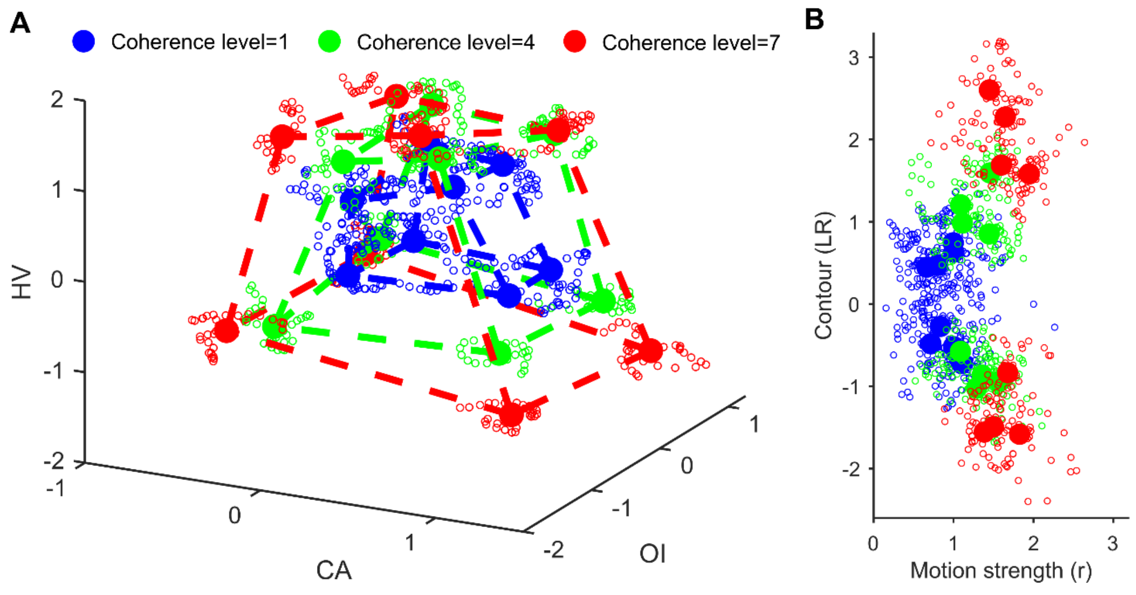

Fig. S8. Neural geometries for different coherence levels. (A) Sensory manifolds in a 3-D space constructed by the three stimulus dimensions for different coherence levels. Vertices on sensory manifolds elicited by different coherence levels showed the geometry of concentric cubes when clusters' centroids (filled circles) at each coherence level were connected by dotted lines. Colors represent different coherence levels. The results for each monkey were shown in Fig. S9 C & D.(B) The projection of neural data onto the LR axis. The vertical axis represents the projections of the neural states to the LR axis. The horizontal axis represents the distance (r) between the origin and the neural states shown in panel A, reflecting the magnitude of the neural population in response to the coherence level. Note that the horizontal axis shown here did not imply its existence in the neural space; it was presented here for illustrative purpose only.

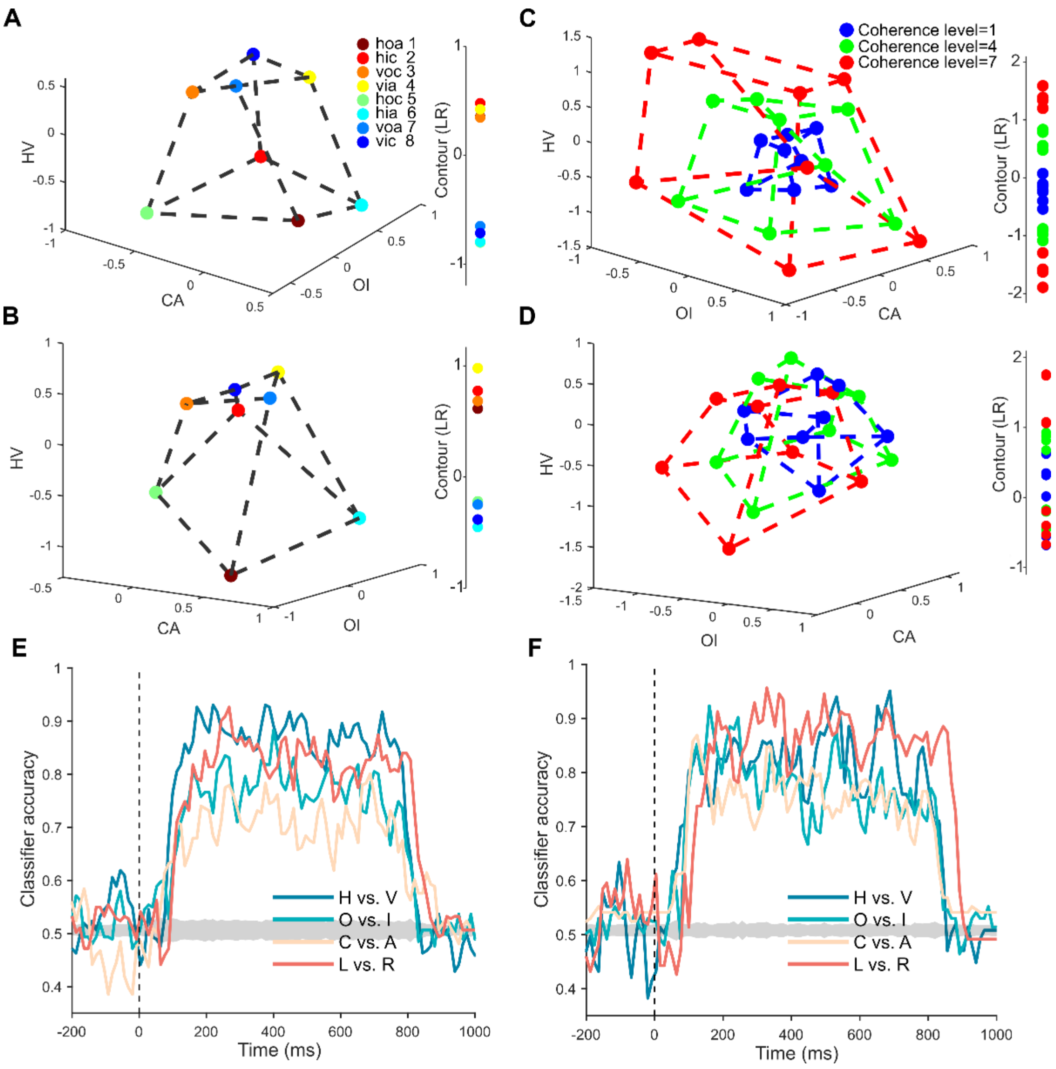

Fig. S9. Sensory manifolds and SVM results for each individual monkey. (A-B) The projection of neural states in the steady phase into the 3-D subspace constructed by the three stimulus dimensions and the 1-D perceptual dimension (i.e., MIC orientations). Only the centroids of the neural states elicited by the 8 stimuli were shown. Panel A and B show the results for Monkey S and W, respectively. (C-D) The effect of motion coherence on sensory and perceptual manifolds, respectively. Panel C and D show the results for Monkey S and W, respectively. (E-F) The time course of SVM classifiers' overall accuracies. Panel E and F show the results for Monkey S and W, respectively.

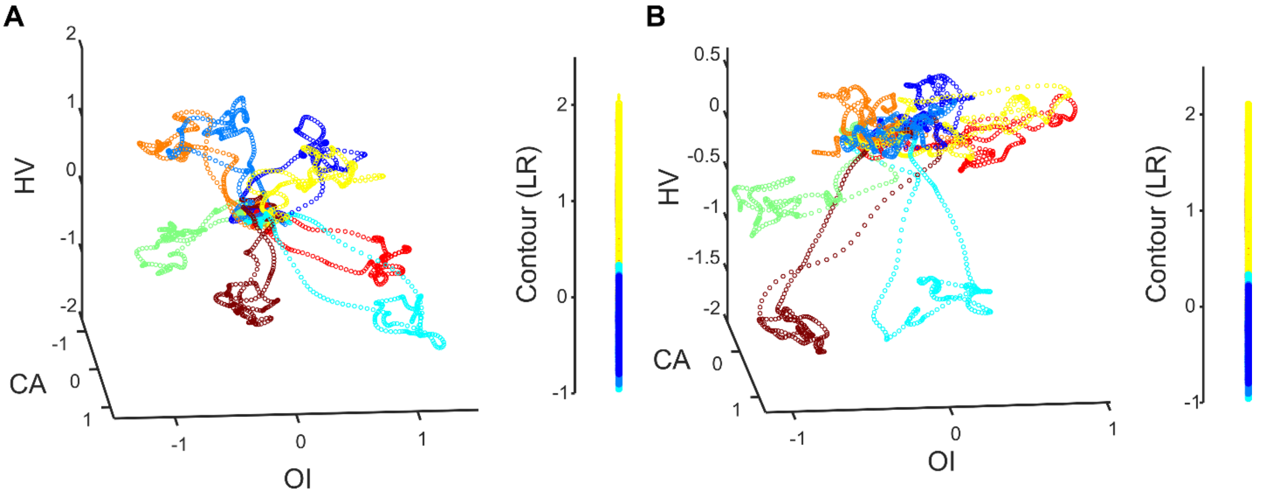

Fig. S10. Neural trajectory at coherence level 7 for each individual monkey.

Panel A and B represent the neural trajectories at a coherence level of 7 for Monkey S and Monkey W, respectively. In each panel, the 3-D subspace (left) depicts the stimulus neural trajectory, and the 1-D subspace (right) depicts the projection of the neural trajectory onto the contour dimension.

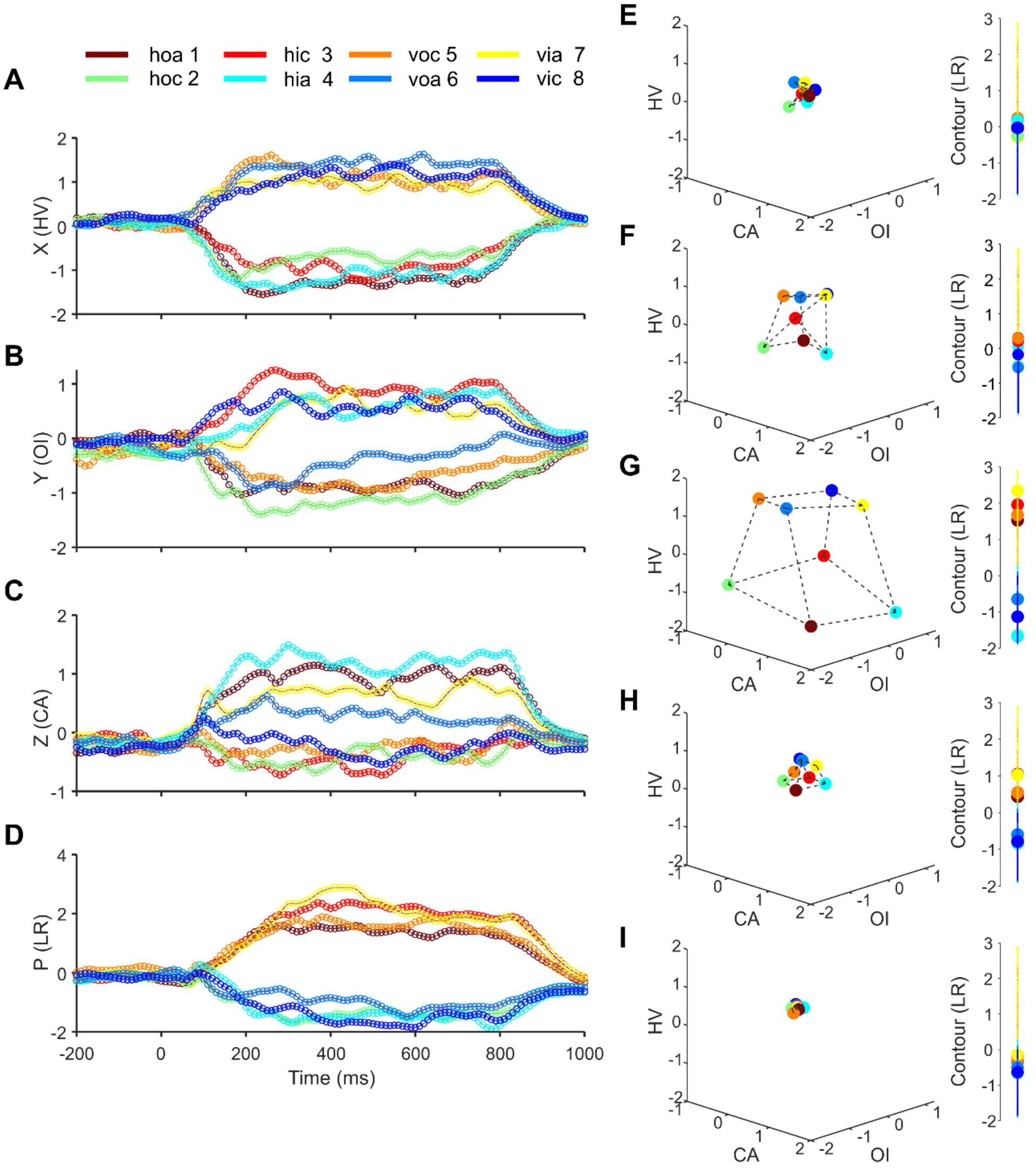

Fig. S11. Neural geometry dynamics in embedded space. (A–D) The time courses of neural states (-200-1000ms) projected to the three sensory axes and one perceptual axis. Colors represent 8 different stimuli. These axes were selective to the corresponding dimension information. (E–I) In the 3-D subspace constructed by the three stimulus dimensions (left) and the 1-D subspace by LR axis (right), the neural manifold at different time points (80, 120, 300, 900, 1000ms, respectively) is shown. Data with a coherence level 7 were used here. After stimulus onset, the manifold expanded gradually from the vicinity of the origin (panel E) to a

cubic shape (panel G). Then, after stimulus offset, the cubic shape collapsed back to the origin (panel I).

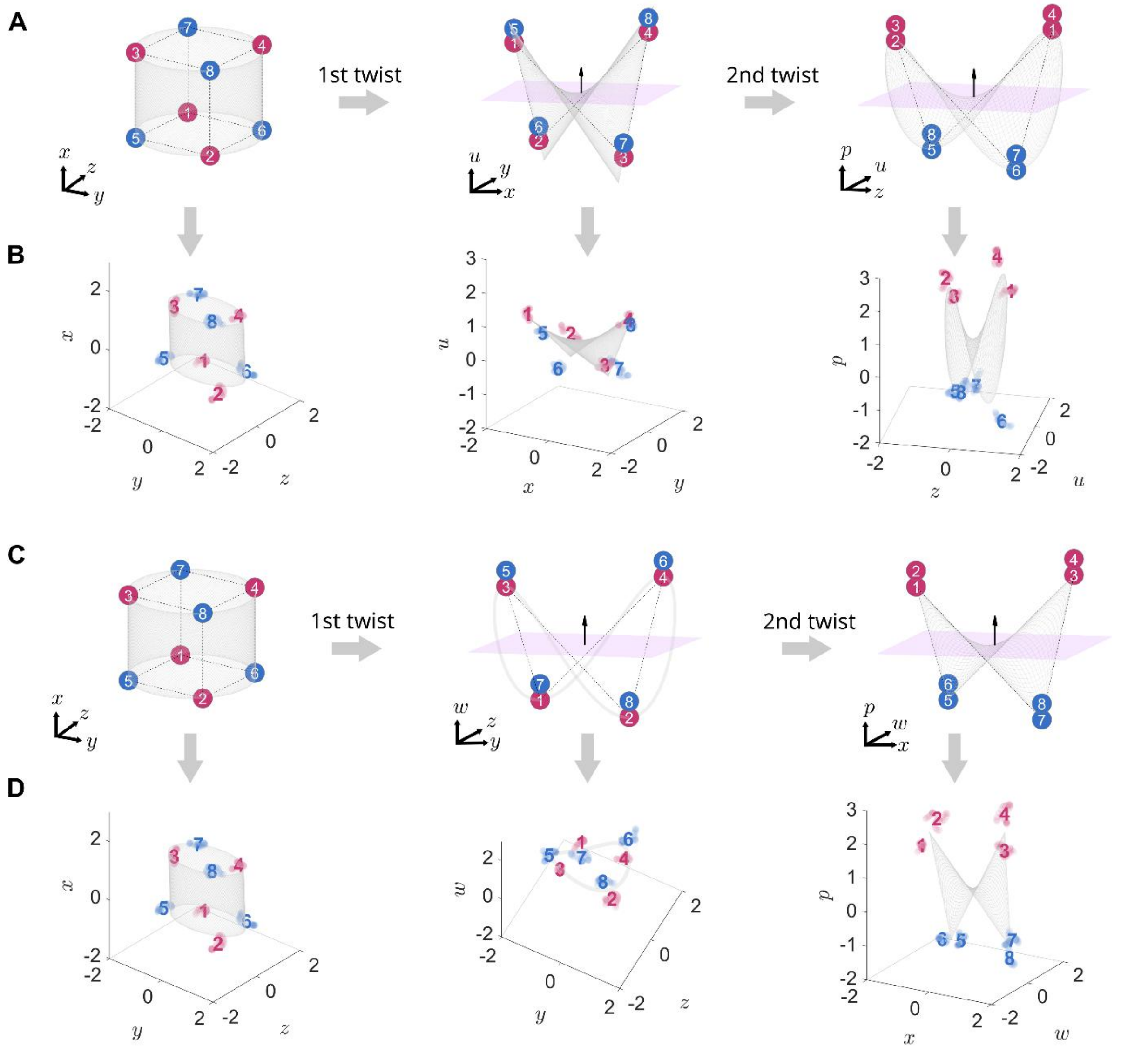

Fig. S12. Two other routes in the double-twisting model. (A) One-twist axis u, equivalent to the logical operation $U = X \oplus Y$ (middle); two-twist axis p, equivalent to the logical operation $P = U \oplus Z$ (right). (B) The neural data were projected to the corresponding subspaces and were fitted to the manifolds presumed by double-twist operations. The existence of the u axis is evident in the middle panel. (C) One-twist axis w, equivalent to the logical operation $W = Y \oplus Z$ (middle); two-twist axis p, equivalent to the logical operation $P = W \oplus X$ (right). (D) Same as panel B except the data are for the route in panel C. The existence of the w axis is evident in the middle panel.

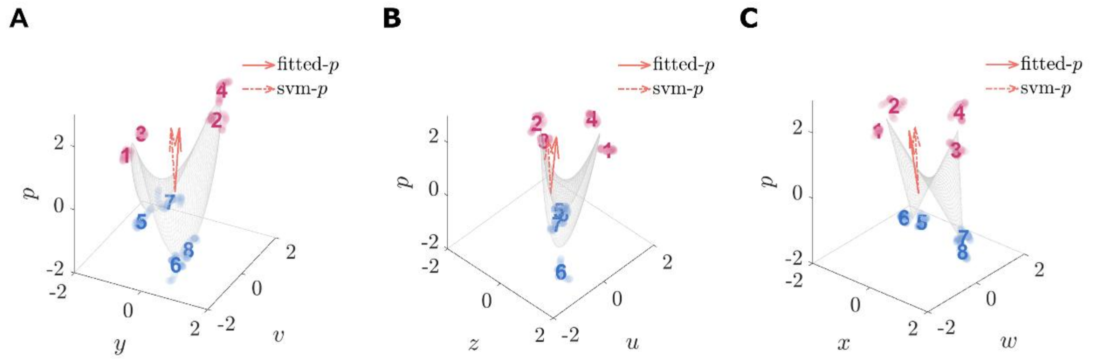

Fig. S13. Comparison between the empirical p axis from the neural data and the theoretical p axis from the double-twist operations. (A)-(C) Three 3-D subspaces incorporating the empirical p axis (i.e., the SVM-p axis) and two other axes were constructed for illustration. By fitting the manifold obtained from double-twist operations to the neural states within these 3-D subspaces using the least-square method, a linear transformation matrix F was generated with one of its column vectors representing the theoretical p axis (i.e., the fitted-p axis). Evidently, in these subspaces, the theoretical p axis was approximately parallel to the empirical p axis. To further quantify this observation, angles between the theoretical and empirical p axes were calculated across all 35 possible 3-D subspaces specified by the double-twist operations, and the angles between these empirical p axes and the theoretical p axis were consistently small (mean = 6.5°, std = 1.8°).

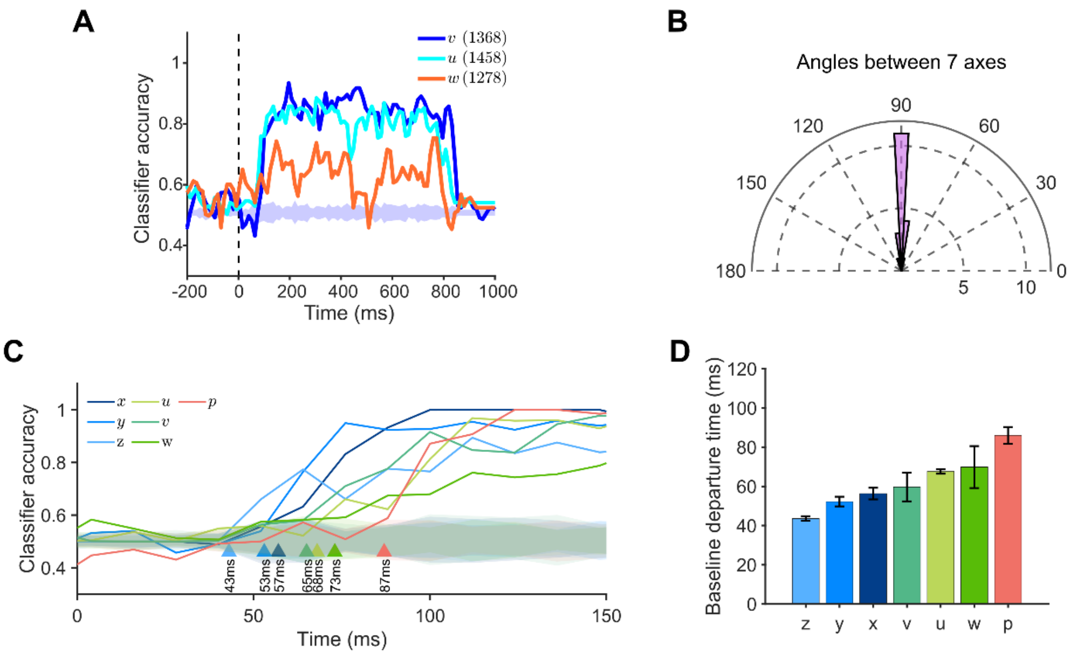

Fig. S14. The accuracies, angles, and emergence timing of the main axes. (A) The linear SVM classification accuracies of the intermediate axes: u, v, w. The corresponding four stimuli, e.g., {1, 3, 6, 8}, that belonged to one class were shown in the parentheses. The shaded area shows the baseline accuracy generated by randomly shuffling the class labels. Note that despite the classification accuracy of the w axis being lower than other axes' accuracies, it was higher than the baseline for most of stimulus presentation time. (B) The distribution of the angles between any pair of the 7 main axes, including the x, y, z and p axes in Fig. 2A and u, v, and w in panel A. All the angles concentrated around 90°. Thus, the 7 main axes were mutually orthogonal. (C) To examine whether the emerging latencies differed significantly across axes, we randomly divided the neural data into training and testing sets 100 times to obtain SVM classification accuracies for each axis. The emerging latency of an axis was defined as the time point where its classification accuracy exceeded the shuffled baseline and remained above it during stimulus presence. Evidently, the latencies for the z, y, and x axes were the shortest (no twist occurred); the v, u, and w axes had intermediate latencies (after 1 twist); and the p axis had the longest latency (after 2 twists). (D) Pairwise t-tests (Bonferroni corrected) showed that the latency differences between any two axes adjacent in the ranking order were significant ($ps < 0.001$), except for the u and w axis difference ($p = 0.08$). Therefore, the ranking order of the

emergence among individual axes aligned with the latency ranking order among the 0-twist, 1-twist, and 2-twist axes reported in the study.

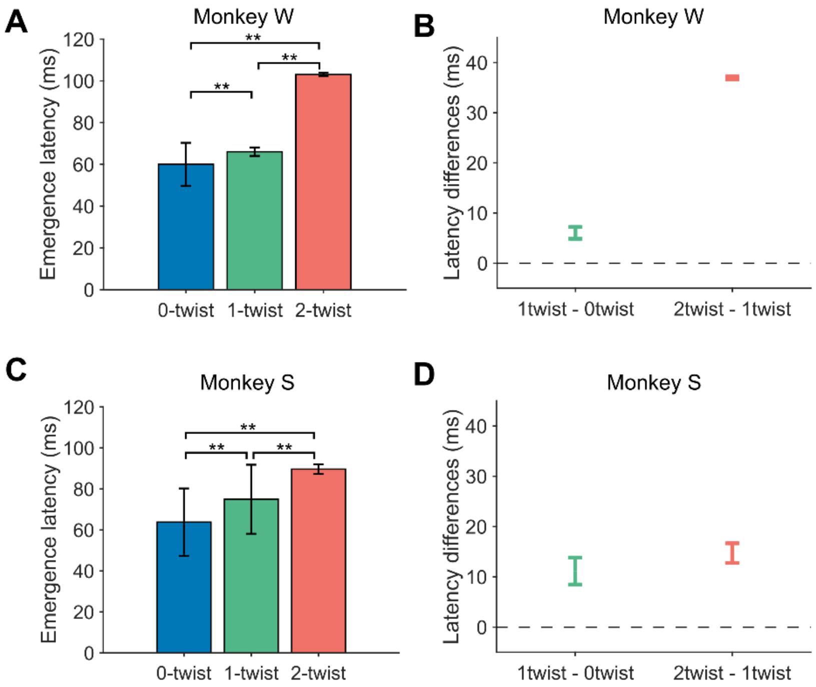

Fig. S15. The emergence timing of the main axes for single monkey.(A) In the data of monkey W, mean emergence latencies of the sensory axes (0-twist, blue; mean=60.0 ms, std=10.3 ms, N=300) is significant smaller than the intermediate axes (1-twist, green; mean=66.1 ms, std=2.0 ms, N=300), and the task-relevant axis (2-twist, red; mean=103.0 ms, std=0.8 ms, N=100). **: bootstrapped t-test p<0.001; error bar: std. (B) The 95% bootstrapped confidence intervals of the latency differences between axes of two different categories in the data of monkey W. The confidence interval of latency differences between the 1-twist and 0-twist axes is [4.9 ms, 7.2 ms], and between the 2-twist and 1-twist axes is [36.7 ms, 37.2 ms], both excluding zero. (C) In the data of monkey S, mean emergence latencies of the sensory axes (0-twist, blue; mean=63.8 ms, std=16.5 ms, N=300) is significant smaller than the intermediate axes (1-twist, green; mean=75.0 ms, std=16.8 ms, N=300), and the task-relevant axis (2-twist, red; mean=89.7 ms, std=2.4 ms, N=100). **: bootstrapped t-test p<0.001; error bar: std. (B) The 95% bootstrapped confidence intervals of the latency differences between axes of two different categories in the data of monkey S. The confidence interval of latency differences between the 1-twist and 0-twist axes is [8.5 ms, 13.8 ms], and between the 2-twist and 1-twist axes is [12.8 ms, 16.6 ms], both excluding zero.

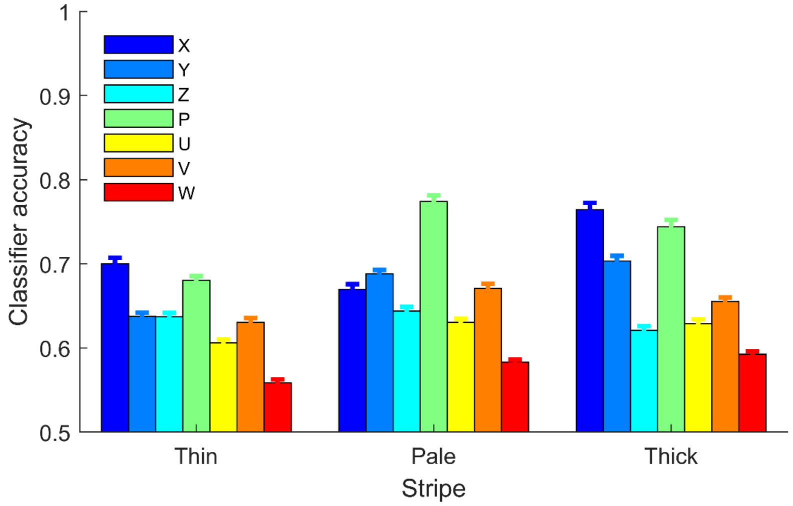

Fig. S16. The representation of the 7 axes in thin, pale, and thick stripes of the V2 area, respectively. In the V2 area, there are three types of cytochrome oxidase (CO) stripes – thin, thick, and pale – each associated with relatively distinct visual processing functions like color, motion and depth perception, spatial orientation and form, respectively. The 93 neurons were divided into three groups according to their respective stripe types: thin (n = 19), thick (n = 39), and pale (n = 35), based on the recording sites in the V2 area (32). SVM analyses identical to those in the study were performed to assess classification accuracies for the 7 axes (each repeated 100 times) in the three stripes. While the classification accuracies were generally lower due to the smaller number of neurons for each stripe, the 7 axes were identified in each of three CO stripes (ps < 0.001), with similar accuracy distributions (rs > 0.7, ps < 0.05). Therefore, the findings in the study likely reflect a general property of V2 neurons.

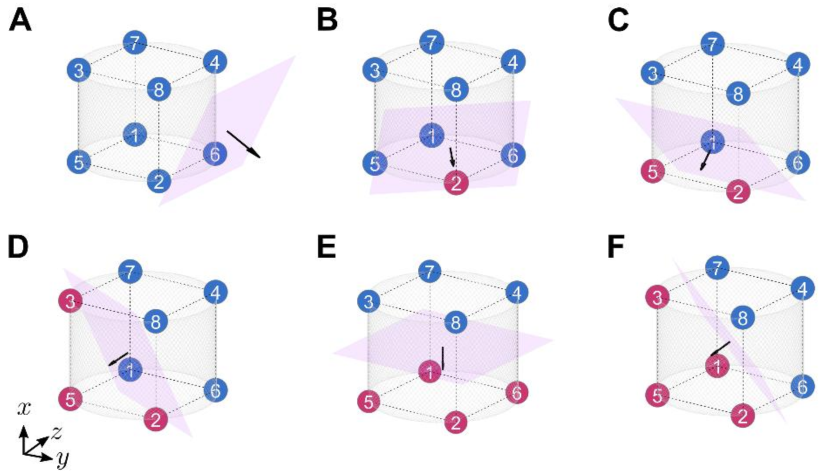

Fig. S17. The number of linear binary classifications in the 3-D stimulus space. A classification is denoted by subsets X versus X^c , and its symmetric classification is X^c versus X. We only consider one side of the symmetry. (A) When $\|X\| = 0$, there is 1 classification. (B) When $\|X\| = 1$, there are 8 classifications for isolating each vertex. (C) When $\|X\| = 2$, there are 12 classifications for isolating each edge. (D) When $\|X\| = 3$, there are 24 classifications for 4 different ways of isolating 3 vertices on 6 faces. (E) When $\|X\| = 4$, in this parallel case, there are 3 classifications for isolating a complete face. (F) When $\|X\| = 4$, in this corner case, there are 4 classifications for isolating a complete corner. Overall, there are $(1 + 8 + 12 + 24 + 3 + 4) \times 2 = 104$ different linear binary classifications.

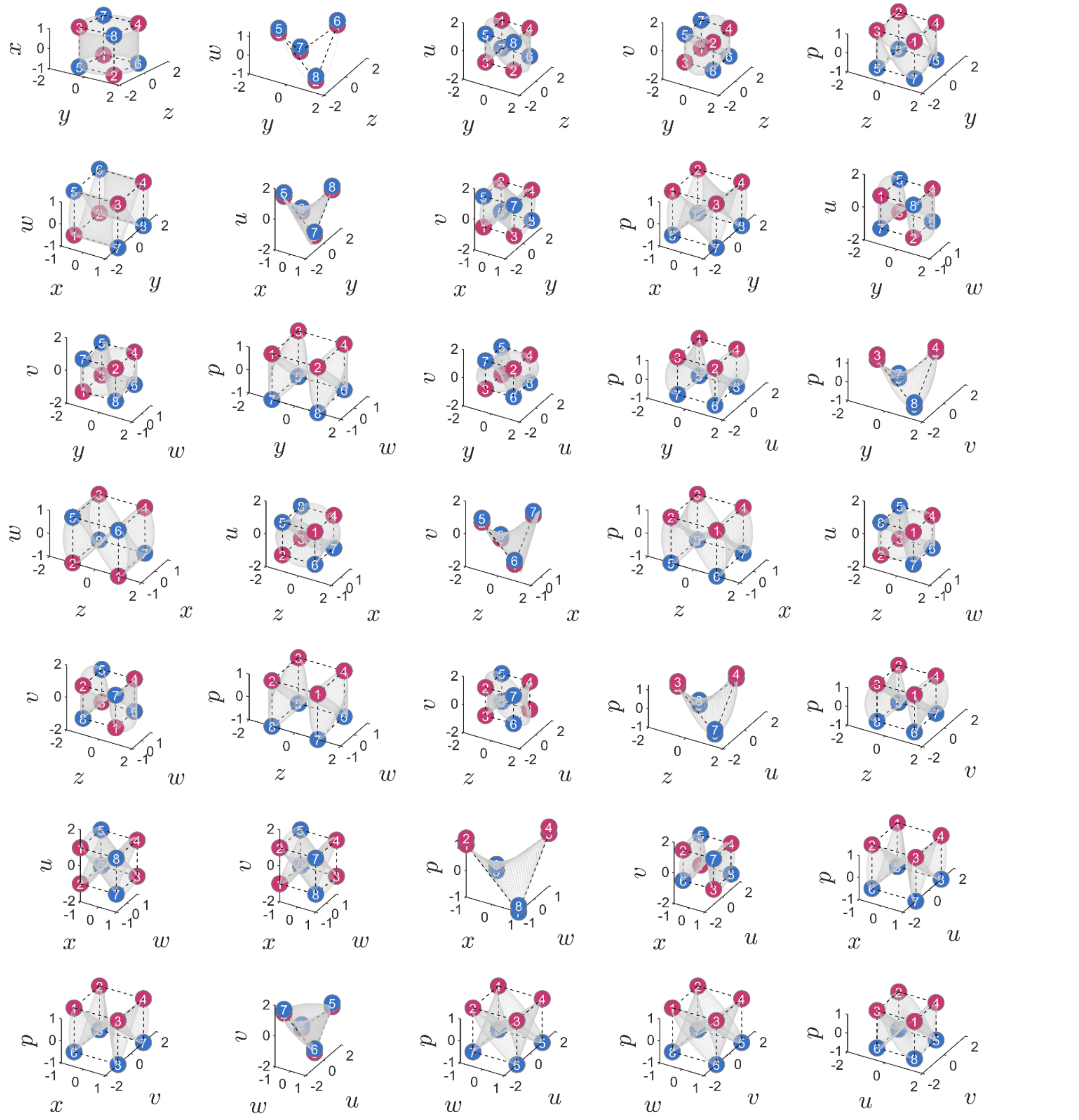

Fig. S18. A complete view of the theoretical perceptual manifold. In the 7-D space generated by double-twist operations, all the linearly inseparable classifications in the 3-D stimulus space becomes linearly separable. By selecting 3 axes of the 7-D space, in total $\binom{7}{3} = 35$ different 3-D subspaces can be constructed. Projections of the perceptual manifold are shown in these subspaces. Each linearly inseparable classification of the stimuli in the stimulus space can find its linearly separable version in one of the 35 subspaces (Table S2). Together, the projections in

the 35 subspaces give a complete view of the theoretical perceptual manifold. Stimuli in the red vertices show the right-tilted contour, and those in the blue vertices show the left-titled contour.

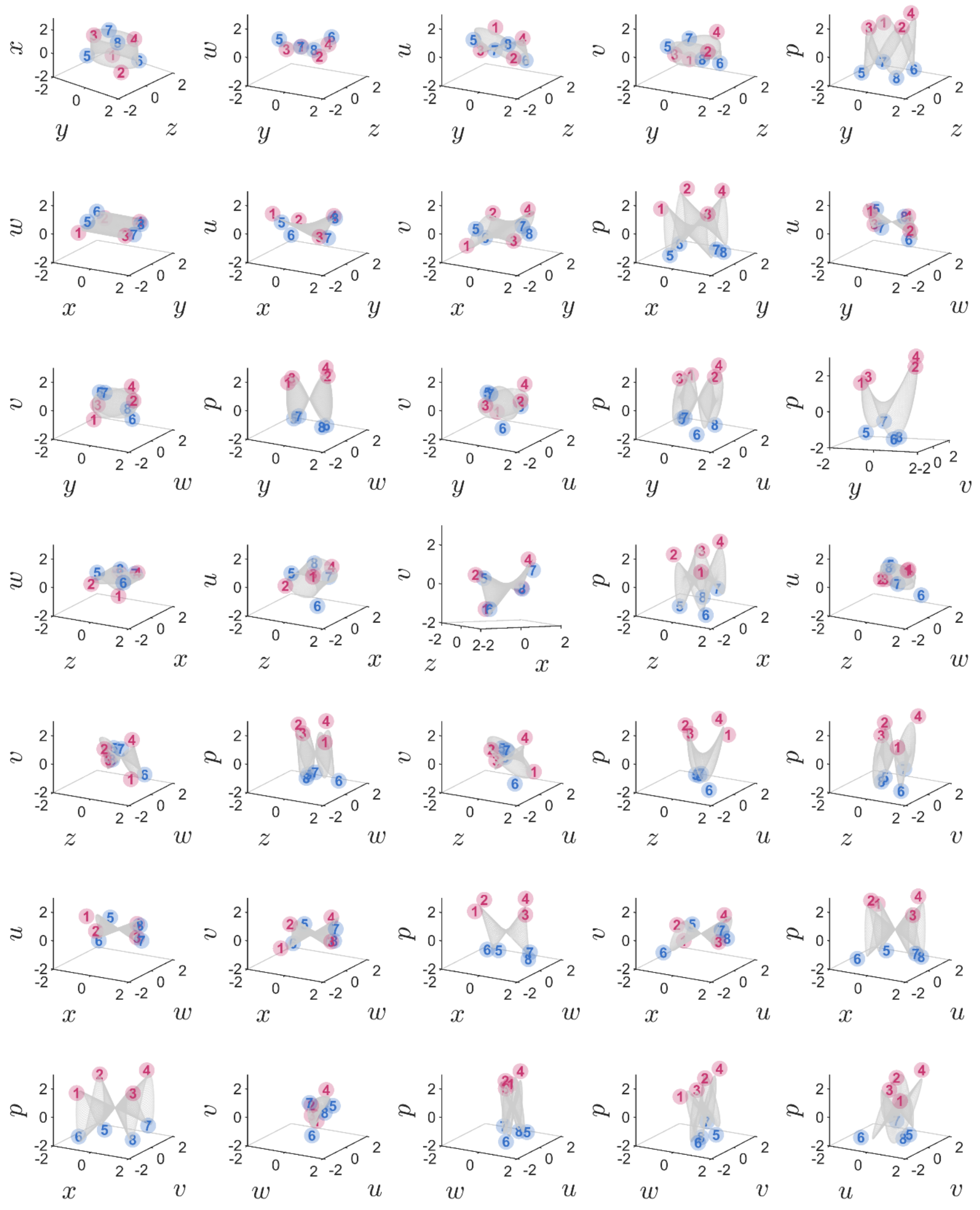

Fig. S19. The neural data in 35 different 3-D perceptual subspaces. Corresponding to the 35 different 3-D subspaces generated by the double-twist operations, we can visualize the neural data in the 35 perceptual subspaces. The subspaces for visualization were created with the 7 main axes found by linear SVMs, because these axes were mutually orthogonal (Fig. S14B). For

subspaces created by axes with high classification accuracies, the consistency between the data and the theoretically derived perceptual manifold was high, for instance, in the y-z-p and y-v-p subspaces. For subspaces created by axes with low classification accuracies, the consistency was relatively low. For instance, because the separation margin of the w axis was short, the data-model consistency in the x-y-w and z-w-u spaces was relatively small, suggesting that the dimension of the w axis might be less important in the perceptual manifold. Nevertheless, we still can visualize the linear separation in these perceptual subspaces. For clarity, only the centers of the point clusters are shown. The red vertices have a right-tilted contour (R), and the blue ones have a left-tilted contour (L).

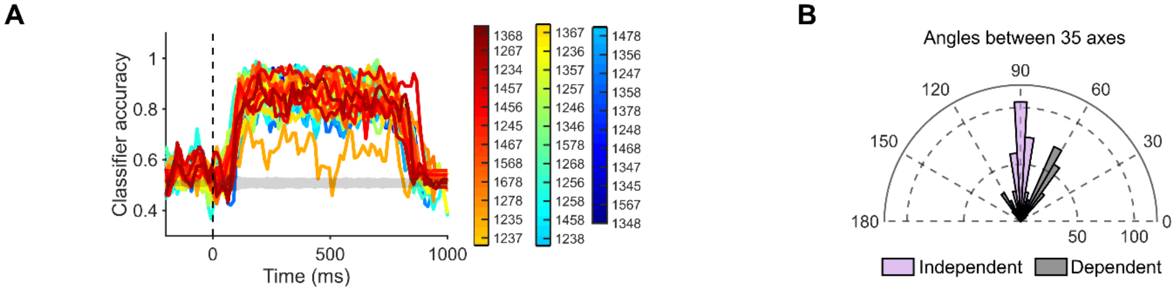

Fig. S20. Existence of dependent and independent classification directions and the angle distribution. (A) The 35 classification accuracies of all 4-element binary classifications. The trajectories are color coded and labeled by the four stimulus conditions in one class. All the classification accuracies are above the baseline (shaded area), except the classification 1278, which is the w axis, was slightly worse as in Fig. S14A. (B) The distribution of the angles between any pair of the 35 classification directions. The angles between classification directions of any two independent classifications are shown in purple, whereas the angles between classification directions of any two dependent classifications are shown in gray. The angles of independent classification directions concentrated around 90°, while the angles of dependent classification directions concentrated around either 60° or 120°.

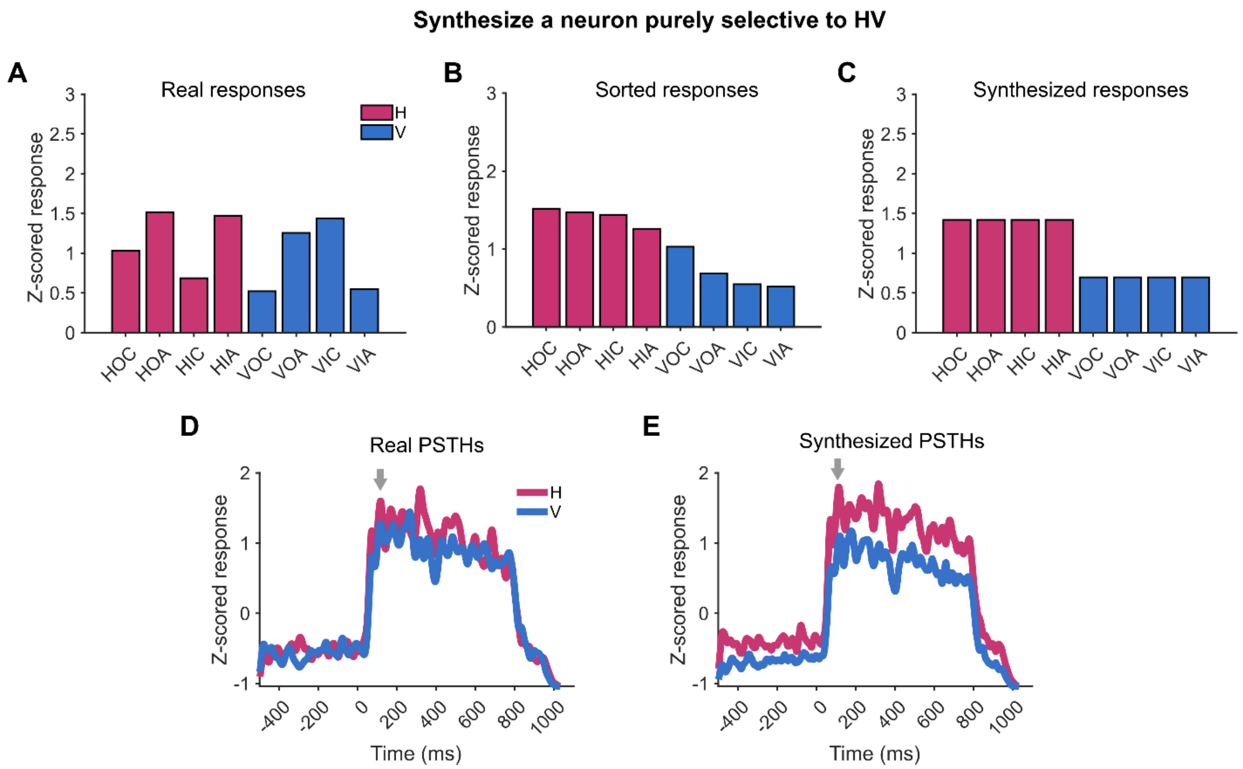

Fig. S21. The procedure of synthesizing neurons with pure selectivity. (A) The activities of each neuron at each time bin were recorded for all the stimuli. The stimuli are labelled by abbreviations, e.g., HOC indicates the horizontal-outward-clockwise stimulus. Here, an example neuron's responses to different stimuli are shown. The responses are colored according to the HV dimension. The purpose was to synthesize an artificial neuron that was purely selective to HV but retaining the variability of the neuron's real responses. The process took two steps: sorting and averaging at each time bin. (B) Step 1: The activities of all stimulus conditions were sorted regardless of the class labels. Then, the first half of the stimulus conditions were relabeled H while the second half were relabeled V. (C) Step 2: The mean activities of each class were reassigned to every stimulus in their respective class. (D) The actual averaged Peri-Stimulus Time Histograms (PSTHs) of the neuron in panel A. This neuron did not show evident selectivity for any stimulus dimension. (E) The averaged PSTHs of the synthesized pure selectivity neuron. The selectivity to HV became clear. Arrows indicate the time bin during which the neuron's activities are shown in panel A, B, and C.

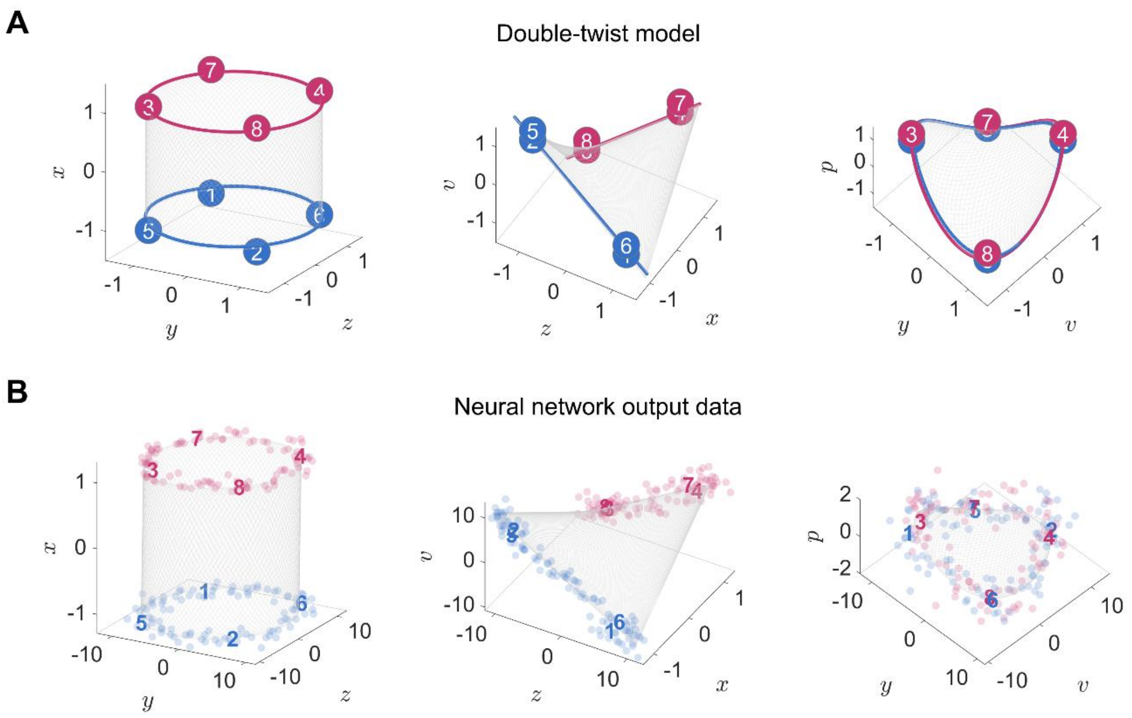

Fig. S22. The comparison between the theoretically-derived perceptual manifold and simulated neural network data. (A) The theoretically-derived perceptual manifold in which the stimuli spanned the entire sheared configuration rings for the vertical and horizontal motion-axis. The continuous stimuli are highlighted in color. Red represents the vertical motion-axis and blue represents horizontal motion-axis. Among the continuous stimuli, 8 stimuli that were tested in the experiment are marked by solid circles. Left to right represents subspaces after 0-twist, 1-twist, and 2-twist operations, respectively. (B) The simulated network data visualized in the subspaces corresponding to panel A. The input data points were sampled from the continuous rings in the stimulus space. Left to right shows the visualization of the output data. The background manifolds were fitted to the data points corresponding to the 8 stimuli in each subspace. The rest data points positioned on the manifolds in a continuous fashion. Comparison between A and B shows close alignment of the manifold formed by the neural network to that predicted by the double-twist operations.

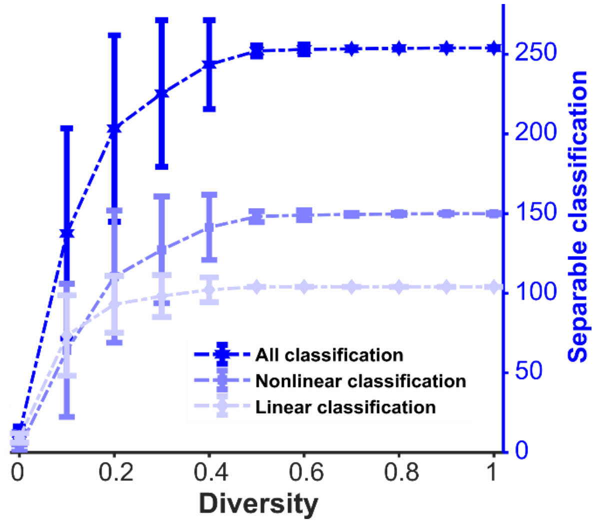

Fig. S23. The number of successful classifications as a function of diversity. Three color curves represent the number of successful classifications as the function of diversity. Among these, dark blue represents all binary classification problems (256 in total), blue represents linearly inseparable binary classification problems in the 3-D stimulus space (152 in total), and light bule represents linearly separable binary classification problems in the 3-D stimulus space (104 in total).

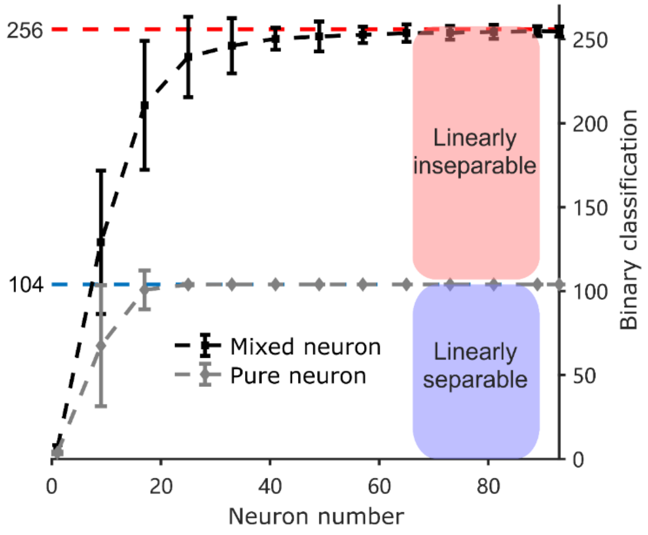

Fig. S24. The number of successful linear classifications increases as a function of neuron population size for both pure selectivity neurons and NMS neurons. In the neural network, the parameter d, representing the diversity in the connection patterns, was set to 0.6. Pure selectivity neurons were defined by their responsiveness solely to a single feature. In creating a 2-layer network similar to that shown in Fig. 6D, yet featuring pure selectivity neurons in the second layer, we adopted a similar approach to specifying connection weights as detailed in the "Connectivity patterns of neural network" section of the Methods, with a critical modification that each neuron's response was determined exclusively by a single feature to ensure pure selectivity. Simulations showed that the network with pure selectivity neurons successfully classified all linearly separable problems (104 instances in total, gray curve) but failed to solve any linearly inseparable problem. In contrast, with the same parameter d (i.e., 0.6), the network with NMS neurons successfully classified both linearly separable and inseparable problems (256 instances in total, dark curve), underscoring the essential role of NMS neurons in tackling such complex problems. Error bar: standard deviation.

A

xor ⊕		
x	y	$x \oplus y$
F	F	F
F	T	T
T	F	T
T	T	F

B

product		
x	y	$x \cdot y$
1.0	1.0	1.0
1.0	-1.0	-1.0
-1.0	1.0	-1.0
-1.0	-1.0	1.0

Fig. S25. The correspondence between XOR and arithmetic product. (A) The truth table of XOR operation on two Boolean variables X and Y. F: False, and T: True. (B) The results of multiplying two real number x and y. The color code indicates the correspondence between -1.0 and the true value, and that between 1.0 and the false value. The last column in panel B aligns with the last column in panel A according to this correspondence.

Table S1. The 104 linearly separable classifications in the stimulus space.

Subspace y-z-x					
∅ vs. 12345678	15 vs. 234678	46 vs. 123578	156 vs. 23478	268 vs. 13457	1235 vs. 4678
1 vs. 2345678	16 vs. 234578	47 vs. 123568	157 vs. 23468	347 vs. 12568	1246 vs. 3578
2 vs. 1345678	17 vs. 234578	48 vs. 123567	167 vs. 23458	348 vs. 12567	1256 vs. 3478
3 vs. 1245678	25 vs. 134678	125 vs. 34678	235 vs. 14678	357 vs. 12468	1347 vs. 2568
4 vs. 1235678	26 vs. 134578	126 vs. 34578	238 vs. 14567	358 vs. 12467	1357 vs. 2468
5 vs. 1234678	28 vs. 134567	135 vs. 24678	246 vs. 13578	378 vs. 12456	1467 vs. 2358
6 vs. 1234578	35 vs. 123678	137 vs. 24568	248 vs. 13567	467 vs. 12358	1567 vs. 2348
7 vs. 1234568	37 vs. 124568	146 vs. 23578	256 vs. 13478	468 vs. 12357	
8 vs. 1234567	38 vs. 124567	147 vs. 23568	258 vs. 13467	478 vs. 12356	

Table S2. The 152 linearly inseparable classifications become linearly separable in new subspaces obtained with the double-twist model.

Subspace y-z-p		Subspace y-z-u		Subspace y-z-v	
12 vs. 345678	234 vs. 15678	14 vs. 235678	237 vs. 14568	57 vs. 123468	247 vs. 13568
13 vs. 245678	567 vs. 12348	58 vs. 123467	267 vs. 13458	68 vs. 123457	257 vs. 13468
23 vs. 145678	568 vs. 12347	67 vs. 123458	367 vs. 12458	136 vs. 24578	368 vs. 12457
24 vs. 135678	578 vs. 12346	145 vs. 23678	458 vs. 12367	138 vs. 24567	457 vs. 12368
123 vs. 45678	678 vs. 12345	148 vs. 23567	1358 vs. 2467	168 vs. 23457	1457 vs. 2368
124 vs. 35678	1234 vs. 5678	158 vs. 23467	1367 vs. 2458	245 vs. 13678	1468 vs. 2357
134 vs. 25678		236 vs. 14578			
**Subspace y-w-p**		**Subspace z-x-w**		**Subspace z-w-v**	
1237 vs. 4568		34 vs. 125678	345 vs. 12678	18 vs. 234567	1238 vs. 4567
1248 vs. 3567		56 vs. 123478	346 vs. 12578	27 vs. 134568	1247 vs. 3568
1345 vs. 2678		78 vs. 123456	356 vs. 12478	36 vs. 124578	1346 vs. 2578
1578 vs. 2346		127 vs. 34568	1258 vs. 3467	45 vs. 123678	1678 vs. 2345
		128 vs. 34567	1267 vs. 3458		
		178 vs. 23456	1456 vs. 2378		

	278 vs. 13456	1478 vs. 2356

Subspace x-y-w	Subspace x-u-p	Subspace w-u-v
456 vs. 12378	1236 vs. 4578	1278 vs. 3456
1257 vs. 3468	1245 vs. 3678	1368 vs. 2457
1268 vs. 3457	1348 vs. 2567	1458 vs. 2367
1356 vs. 2478	1568 vs. 2357	
1378 vs. 2456		

Movie S1. Eight MIC stimuli.

Eight MIC stimuli were used in this study, hoa, hic, voc, via, hoc, hia, voa, and vic respectively.

Movie S2. SVM accuracies and dynamic trajectories.

This movie showed **dynamic trajectories of sensory and perception axes corresponding to their SVM accuracies.**

REFERENCES AND NOTES

1. I. Biederman, Recognition-by-components: A theory of human image understanding. *Psychol. Rev.* **94**, 115–147 (1987).

2. S. Chung, D. D. Lee, H. Sompolinsky, Classification and geometry of general perceptual manifolds. *Phys. Rev. X* **8**, 031003 (2018).

3. J. J. DiCarlo, D. Zoccolan, N. C. Rust, How does the brain solve visual object recognition? *Neuron* **73**, 415–434 (2012).

4. M. L. Minsky, S. Papert, *Perceptrons: An Introduction to Computational Geometry, Expanded Ed.* (MIT Press, 1969).

5. S. Fusi, E. K. Miller, M. Rigotti, Why neurons mix: High dimensionality for higher cognition. *Curr. Opin. Neurobiol.* **37**, 66–74 (2016).

6. B. Schölkopf, A. J. Smola, *Learning with Kernels: Support Vector Machines, Regularization, Optimization, and Beyond* (MIT Press, 2002).

7. C. K. I. Williams, C. E. Rasmussen, *Gaussian Processes for Machine Learning* (MIT Press, 2006).

8. H. S. Seung, D. D. Lee, The manifold ways of perception. *Science* **290**, 2268–2269 (2000).

9. J. J. DiCarlo, D. D. Cox, Untangling invariant object recognition. *Trends Cogn. Sci.* **11**, 333–341 (2007).

10. S. Chung, L. F. Abbott, Neural population geometry: An approach for understanding biological and artificial neural networks. *Curr. Opin. Neurobiol.* **70**, 137–144 (2021).

11. E. Froudarakis, U. Cohen, M. Diamantaki, E. Y. Walker, J. Reimer, P. Berens, H. Sompolinsky, A. S. Tolias, Object manifold geometry across the mouse cortical visual hierarchy. bioRxiv 258798 [Preprint] (2020).

12. L. Dyballa, A. M. Rudzite, M. S. Hoseini, M. Thapa, M. P. Stryker, G. D. Field, S. W. Zucker, Population encoding of stimulus features along the visual hierarchy. *Proc. Natl. Acad. Sci. U.S.A.* **121**, e2317773121 (2024).

13. Y. Xie, P. Hu, J. Li, J. Chen, W. Song, X. J. Wang, T. Yang, S. Dehaene, S. Tang, B. Min, L. Wang, Geometry of sequence working memory in macaque prefrontal cortex. *Science* **375**, 632–639 (2022).

14. L. She, M. K. Benna, Y. Shi, S. Fusi, D. Y. Tsao, Temporal multiplexing of perception and memory codes in IT cortex. *Nature* **629**, 861–868 (2024).

15. G. Okazawa, C. E. Hatch, A. Mancoo, C. K. Machens, R. Kiani, Representational geometry of perceptual decisions in the monkey parietal cortex. *Cell* **184**, 3748–3761.e18 (2021).

16. T. Flesch, K. Juechems, T. Dumbalska, A. Saxe, C. Summerfield, Orthogonal representations for robust context-dependent task performance in brains and neural networks. *Neuron* **110**, 1258–1270.e11 (2022).

17. M. Genkin, K. V. Shenoy, C. Chandrasekaran, T. A. Engel, The dynamics and geometry of choice in the premotor cortex. *Nature* **645**, 168–176 (2025).

18. R. J. Gardner, E. Hermansen, M. Pachitariu, Y. Burak, N. A. Baas, B. A. Dunn, M.-B. Moser, E. I. Moser, Toroidal topology of population activity in grid cells. *Nature* **602**, 123–128 (2022).

19. I. I. C. Low, L. M. Giocomo, A. H. Williams, Remapping in a recurrent neural network model of navigation and context inference. bioRxiv 525596 [Preprint] (2023).

20. M. M. Churchland, J. P. Cunningham, M. T. Kaufman, J. D. Foster, P. Nuyujukian, S. I. Ryu, K. V. Shenoy, Neural population dynamics during reaching. *Nature* **487**, 51–56 (2012).

21. J. A. Gallego, M. G. Perich, L. E. Miller, S. A. Solla, Neural manifolds for the control of movement. *Neuron* **94**, 978–984 (2017).

22. J. A. Gallego, M. G. Perich, S. N. Naufel, C. Ethier, S. A. Solla, L. E. Miller, Cortical population activity within a preserved neural manifold underlies multiple motor behaviors. *Nat. Commun.* **9**, 4233 (2018).

23. A. A. Russo, R. Khajeh, S. R. Bittner, S. M. Perkins, J. P. Cunningham, L. F. Abbott, M. M. Churchland, Neural trajectories in the supplementary motor area and motor cortex exhibit distinct geometries, compatible with different classes of computation. *Neuron* **107**, 745–758.e6 (2020).

24. V. L. Marcar, S. E. Raiguel, D. Xiao, G. A. Orban, Processing of kinetically defined boundaries in areas V1 and V2 of the macaque monkey. *J. Neurophysiol.* **84**, 2786–2798 (2000).

25. S. G. Mysore, R. Vogels, S. E. Raiguel, G. A. Orban, Processing of kinetic boundaries in macaque V4. *J. Neurophysiol.* **95**, 1864–1880 (2006).

26. M. Chen, P. Li, S. Zhu, C. Han, H. Xu, Y. Fang, J. Hu, A. W. Roe, H. D. Lu, An orientation map for motion boundaries in macaque V2. *Cereb. Cortex* **26**, 279–287 (2016).

27. I. Mareschal, C. L. Baker Jr., A cortical locus for the processing of contrast-defined contours. *Nat. Neurosci.* **1**, 150–154 (1998).

28. R. von der Heydt, H. Zhou, H. S. Friedman, Representation of stereoscopic edges in monkey visual cortex. *Vision Res.* **40**, 1955–1967 (2000).

29. C. E. Bredfeldt, B. G. Cumming, A simple account of cyclopean edge responses in macaque V2. *J. Neurosci.* **26**, 7581–7596 (2006).

30. Y. El-Shamayleh, J. A. Movshon, Neuronal responses to texture-defined form in macaque visual area V2. *J. Neurosci.* **31**, 8543–8555 (2011).

31. H. D. Lu, G. Chen, H. Tanigawa, A. W. Roe, A motion direction map in macaque V2. *Neuron* **68**, 1002–1013 (2010).

32. H. Ma, P. Li, J. Hu, X. Cai, Q. Song, H. D. Lu, Processing of motion-boundary orientation in macaque V2. *eLife* **10**, e67369 (2021).

33. O. Braddick, Segmentation versus integration in visual motion processing. *Trends Neurosci.* **16**, 263–268 (1993).

34. V. A. F. Lamme, The neurophysiology of figure-ground segregation in primary visual cortex. *J. Neurosci.* **15**, 1605–1615 (1995).

35. J. Hu, H. Ma, S. Zhu, P. Li, H. Xu, Y. Fang, M. Chen, C. Han, C. Fang, X. Cai, K. Yan, H. D. Lu, Visual motion processing in macaque V2. *Cell Rep.* **25**, 157–167.e5 (2018).

36. M. Rigotti, O. Barak, M. R. Warden, X. J. Wang, N. D. Daw, E. K. Miller, S. Fusi, The importance of mixed selectivity in complex cognitive tasks. *Nature* **497**, 585–590 (2013).

37. M. T. Kaufman, M. K. Benna, M. Rigotti, F. Stefanini, S. Fusi, A. K. Churchland, The implications of categorical and category-free mixed selectivity on representational geometries. *Curr. Opin. Neurobiol.* **77**, 102644 (2022).

38. K. M. Tye, E. K. Miller, F. H. Taschbach, M. K. Benna, M. Rigotti, S. Fusi, Mixed selectivity: Cellular computations for complexity. *Neuron* **112**, 2289–2303 (2024).

39. G. Cybenko, Approximation by superpositions of a sigmoidal function. *Math. Control Signals Syst.* **2**, 303–314 (1989).

40. Y. LeCun, Y. Bengio, G. Hinton, Deep learning. *Nature* **521**, 436–444 (2015).

41. W. Dang, R. J. Jaffe, X. L. Qi, C. Constantinidis, Emergence of nonlinear mixed selectivity in prefrontal cortex after training. *J. Neurosci.* **41**, 7420–7434 (2021).

42. R. Nogueira, C. C. Rodgers, R. M. Bruno, S. Fusi, The geometry of cortical representations of touch in rodents. *Nat. Neurosci.* **26**, 239–250 (2023).

43. A. Rutherford, *ANOVA and ANCOVA: A GLM approach* (John Wiley & Sons, 2011).

44. M. Rigotti, D. B. D. Rubin, X.-J. Wang, S. Fusi, Internal representation of task rules by recurrent dynamics: The importance of the diversity of neural responses. *Front. Comput. Neurosci.* **4**, 24 (2010).

45. N. Kriegeskorte, X. X. Wei, Neural tuning and representational geometry. *Nat. Rev. Neurosci.* **22**, 703–718 (2021).

46. R. Gast, S. A. Solla, A. Kennedy, Neural heterogeneity controls computations in spiking neural networks. *Proc. Natl. Acad. Sci. U.S.A.* **121**, e2311885121 (2024).

47. P. Bao, L. She, M. McGill, D. Y. Tsao, A map of object space in primate inferotemporal cortex. *Nature* **583**, 103–108 (2020).

48. M. G. Perich, S. Conti, M. Badi, A. Bogaard, B. Barra, S. Wurth, J. Block, G. Courtine, S. Micera, M. Capogrosso, T. Milekovic, Motor cortical dynamics are shaped by multiple distinct subspaces during naturalistic behavior. bioRxiv 228767 [Preprint] (2020).

49. W. J. Johnston, S. E. Palmer, D. J. Freedman, Nonlinear mixed selectivity supports reliable neural computation. *PLOS Comput. Biol.* **16**, e1007544 (2020).

50. M. Jazayeri, S. Ostojic, Interpreting neural computations by examining intrinsic and embedding dimensionality of neural activity. *Curr. Opin. Neurobiol.* **70**, 113–120 (2021).

51. C. Langdon, M. Genkin, T. A. Engel, A unifying perspective on neural manifolds and circuits for cognition. *Nat. Rev. Neurosci.* **24**, 363–377 (2023).

52. N. Caporale, Y. Dan, Spike timing–dependent plasticity: A Hebbian learning rule. *Annu. Rev. Neurosci.* **31**, 25–46 (2008).

53. J. A. Bullinaria, Understanding the emergence of modularity in neural systems. *Cogn. Sci.* **31**, 673–695 (2007).

54. G. W. Lindsay, M. Rigotti, M. R. Warden, E. K. Miller, S. Fusi, Hebbian learning in a random network captures selectivity properties of the prefrontal cortex. *J. Neurosci.* **37**, 11021–11036 (2017).

55. M. Weigand, F. Sartori, H. Cuntz, Universal transition from unstructured to structured neural maps. *Proc. Natl. Acad. Sci. U.S.A.* **114**, E4057–E4064 (2017).

56. O. J. Hénaff, Y. Bai, J. A. Charlton, I. Nauhaus, E. P. Simoncelli, R. L. T. Goris, Primary visual cortex straightens natural video trajectories. *Nat. Commun.* **12**, 5982 (2021).

57. K. Rajan, L. F. Abbott, H. Sompolinsky, Stimulus-dependent suppression of chaos in recurrent neural networks. *Phys. Rev. E* **82**, 011903 (2010).

58. S. Ganguli, H. Sompolinsky, Compressed sensing, sparsity, and dimensionality in neuronal information processing and data analysis. *Annu. Rev. Neurosci.* **35**, 485–508 (2012).

59. M. Farrell, S. Recanatesi, T. Moore, G. Lajoie, E. Shea-Brown, Gradient-based learning drives robust representations in recurrent neural networks by balancing compression and expansion. *Nat. Mach. Intell.* **4**, 564–573 (2022).

60. S. Bernardi, M. K. Benna, M. Rigotti, J. Munuera, S. Fusi, C. D. Salzman, The geometry of abstraction in the hippocampus and prefrontal cortex. *Cell* **183**, 954–967.e21 (2020).

61. C. Stringer, M. Pachitariu, N. Steinmetz, M. Carandini, K. D. Harris, High-dimensional geometry of population responses in visual cortex. *Nature* **571**, 361–365 (2019).

62. B. Lin, N. Kriegeskorte, The topology and geometry of neural representations. *Proc. Natl. Acad. Sci. U.S.A.* **121**, e2317881121 (2024).

63. D. Mendoza-Halliday, S. Torres, J. Martinez-Trujillo, Sharp emergence of feature-selective sustained activity along the dorsal visual pathway. *Nat. Neurosci.* **17**, 1255–1262 (2014).

64. J. Huang, T. Wang, W. Dai, Y. Li, Y. Yang, Y. Zhang, Y. Wu, T. Zhou, D. Xing, Neuronal representation of visual working memory content in the primate primary visual cortex. *Sci. Adv.* **10**, eadk3953 (2024).

65. V. Mante, D. Sussillo, K. V. Shenoy, W. T. Newsome, Context-dependent computation by recurrent dynamics in prefrontal cortex. *Nature* **503**, 78–84 (2013).

66. C. J. Cueva, A. Ardalan, M. Tsodyks, N. Qian, Recurrent neural network models for working memory of continuous variables: Activity manifolds, connectivity patterns, and dynamic codes. arXiv:2111.01275 [q-bio.NC] (2021).

67. N. J. Priebe, C. R. Cassanello, S. G. Lisberger, The neural representation of speed in macaque area MT/V5. *J. Neurosci.* **23**, 5650–5661 (2003).

68. M. A. Smith, N. J. Majaj, J. A. Movshon, Dynamics of motion signaling by neurons in macaque area MT. *Nat. Neurosci.* **8**, 220–228 (2005).

69. G. F. Elsayed, A. H. Lara, M. T. Kaufman, M. M. Churchland, J. P. Cunningham, Reorganization between preparatory and movement population responses in motor cortex. *Nat. Commun.* **7**, 13239 (2016).

70. D. Widdows, T. Cohen, Reasoning with vectors: A continuous model for fast robust inference. *Log. J. IGPL* **23**, 141–173 (2015).

71. M. E. Muller, A note on a method for generating points uniformly on *n*-dimensional spheres. *Commun. ACM* **2**, 19–20 (1959).

72. D. E. Knuth, *The Art of Computer Programming, Vol. 2: Seminumerical Algorithms* (Addison-Wesley, 1973).

73. N. Cristianini, J. Shawe-Taylor, *An Introduction to Support Vector Machines and Other Kernel-Based Learning Methods* (Cambridge Univ. Press, 2000).

74. C. Libedinsky, Comparing representations and computations in single neurons versus neural networks. *Trends Cogn. Sci.* **27**, 517–527 (2023).
